# Supplementary material for: Establishing a taxonomy of potential hazards associated with communicating medical science in the age of disinformation
Source: BMJ Open. 2020 Jul 5;10(7):e035626. doi: 10.1136/bmjopen-2019-035626 (PMC7342820; doi:10.1136/bmjopen-2019-035626)
Supplement: Supplementary data [file bmjopen-2019-035626supp001.pdf]

* Required Information

Experiences in public communication of medical science & health

This survey is part of a research project that aims to better understand the experiences of medical professionals, scientific researchers, patients advocates, health journalists, & policy makers in communicating health issues to the public. It consists of 20 short questions, & should take 5-10 minutes to complete. All responses are anonymous, and their content will be used for research purposes only. If you might be willing to contribute more about your experiences we invite you to inform us of this on the final page of the survey. For any questions, please e-mail d.r.grimes@qub.ac.uk

*** 1. Please select that which best describes your primary role (Select one option)**

- ◯ Medical Professional
- ◯ Scientific Communications
- ◯ Scientist / Researcher
- ◯ Health Journalism
- ◯ Patient Advocate
- ◯ Health Policy
- ◯ Other (Please specify) __________

*** 2. Please specify your gender (Select one option)**

- ◯ Female
- ◯ Non-Binary
- ◯ Male
- ◯ Prefer not to say

*** 3. Please indicate which of the following organsiations you are professionally affiliated with that are directly relevant to your outreach efforts.**

- ☐ University / research institute
- ☐ Media outlet
- ☐ Medical institute
- ☐ Political grouping
- ☐ NGO / Charity
- ☐ Non-affiliated (Patient Advocate / independent)
- ☐ Other (Please specify) ______________

*** 4. Please select the topic or topics you frequently communicate about with the public. Please note that this includes topics you might debunk or criticize. Select all that apply.**

- ☐ Vaccination
- ☐ Alternative Medicine
- ☐ Medicine in media
- ☐ Dietary Science
- ☐ Pseudoscience / scams
- ☐ Medical science
- ☐ Cancer
- ☐ Chronic illness
- ☐ Bioethics
- ☐ Health Policy
- ☐ Infectious diseases
- ☐ Interpretation of research
- ☐ Reproductive / Sexual Health
- ☐ Pharmacology / drugs
- ☐ Gender issues
- ☐ Patient Advocacy
- ☐ Psychological issues
- ☐ Genetics
- ☐ Other (Please specify) ______________

*** 5. What media do you use for public engagement? Select all that apply.**

- ☐ Television (appearances)
- ☐ Television (advising / quoted)
- ☐ Radio (appearances)
- ☐ Radio (advising / quoted)
- ☐ Newspapers / Magazines (Author)
- ☐ Newspapers / Magazines (advising / quoted)
- ☐ Books (Author)
- ☐ Books (Advising / quoted)
- ☐ Public talks
- ☐ Podcasts
- ☐ Facebook
- ☐ Twitter
- ☐ Instagram
- ☐ Youtube
- ☐ Blogging
- ☐ Other (Please specify) _______________

*** 6. Approximately how long have you been communicating medical issues to the public? Please round to the nearest whole year**
(Enter a value between 0 and 120)

____

*** 7. Please select what best describes the extent of your public engagement (Select one option)**

- ○ I am heavily involved in public outreach
- ○ I am considerably involved in public outreach
- ○ I am involved in public outreach
- ○ I am lightly involved in public outreach
- ○ My vocation is media or outreach centred
- ○ Other (Please specify) ___________

For any queries, please contact Dr. David Robert Grimes at d.r.grimes@qub.ac.uk or davidrobert.grimes@oncology.ox.ac.uk

Grimes DR, *et al. BMJ Open* 2020; 10:e035626. doi: 10.1136/bmjopen-2019-035626

Experiences in public communication of medical science & health

Part II: Engagement experiences

8. In your opinion, how frequently do the following sentiments apply to your experiences of public engagement?

	Always	Frequently	Sometimes	Infrequently	Never	Unsure
*(a) I feel my efforts contribute to public understanding	○	○	○	○	○	○
*(b) I find engagement prompts constructive and respectful discussion	○	○	○	○	○	○
*(c) Engagement is mutually informative	○	○	○	○	○	○
*(d) My contributions are welcomed & appreciated	○	○	○	○	○	○
*(e) Engagement increases my personal understanding	○	○	○	○	○	○
*(f) I feel I correct some misconceptions	○	○	○	○	○	○
*(g) I find my engagement changes minds & informs	○	○	○	○	○	○
*(h) I consider outreach personally rewarding	○	○	○	○	○	○
*(i) My efforts feel futile	○	○	○	○	○	○
*(j) Engagement takes a toll on my mental health & well-being	○	○	○	○	○	○
*(k) I find my efforts are taken in good faith by others	○	○	○	○	○	○

*** 9. Please indicate whether you have experienced any of the following negative experiences during your engagement efforts. Select all that apply. Note that for the purposes of this survey, vexatious complaints are defined as those raised chiefly to harass or intimidate.**

- ☐ Personal abuse
- ☐ Personal smears
- ☐ Professional smears
- ☐ Physical threats
- ☐ Threats of sexual violence
- ☐ Harassment from individuals
- ☐ Coordinated harassment from specific groups
- ☐ Questioning of one's motivations
- ☐ Other (Please specify) _______________
- ☐ Sexist or gender-specific abuse
- ☐ Racist of ethnic-specific abuse
- ☐ Intimidation attempts
- ☐ Vexatious legal threats
- ☐ Vexatious complaints to employers
- ☐ Vexatious complaints to professional bodies
- ☐ Persistent unwanted communication
- ☐ No negative experiences

*** 10. How would you best describe your feelings about your outreach experiences to date? (Select one option)**

- ○ Largely rewarding
- ○ Rewarding
- ○ Mixed
- ○ Not very rewarding
- ○ Not at all rewarding
- ○ Other (Please specify) ___________

For any queries, please contact Dr. David Robert Grimes at d.r.grimes@qub.ac.uk or davidrobert.grimes@oncology.ox.ac.uk

Experiences in public communication of medical science & health

*** 11. If you have been the recipient of abuse or personal / professional smears stemming from your engagement, please indicate which of the following you've experienced (check all that apply)**

- ☐ Aggressive or intimidating comments
- ☐ Insults based on appearance, race, or gender
- ☐ Malicious comments about one's motivations / allegations of 'corruption' or 'shilling'
- ☐ Malacious comments about one's personal or sexual conduct
- ☐ Other (Please specify) _______________
- ☐ Assertions that you are dishonest or deliberately misleading
- ☐ Insults about your professional competence or intelligence
- ☐ I have not experienced any of the above

*** 12. Please indicate whether you have been subjected to any of the following (check all that apply)**

- ☐ Threats or implications of physical violence
- ☐ Physical violence or intimidation
- ☐ Spreading of malicious rumours
- ☐ Repeated unwanted communications
- ☐ Other (Please specify) _______________
- ☐ Persistent trolling / harassment by an individual
- ☐ Persistent trolling / harassment by a group
- ☐ None of the above

*** 13. If you have been the victim of targeted abuse for your outreach work by a group or community, what best describes that grouping? Choose all that apply if relevant**

- ☐ Anti-vaccine groups
- ☐ Dietary advocates
- ☐ Chronic illness groups
- ☐ Anti-fluoride groups
- ☐ Electromagnetic hypersensitivity groups
- ☐ Other (Please elaborate as appropriate) _______________
- ☐ Religious groupings
- ☐ Alternative medicine advocates
- ☐ "Wellness" groups
- ☐ Autism-focused groups
- ☐ I have not encountered this

*** 14. Please indicate whether you have experienced any of the following. Note that for the purposes of this survey, vexatious complaints are defined as those raised chiefly to harass or intimidate.**

- ☐ Vexatious complaint to employer or institution - complaint dismissed without investigation
- ☐ Vexatious complaint to employer or institution - required investigation
- ☐ Vexatious complaint to a professional body - complaint dismissed without investigation
- ☐ Vexatious complaint to a professional body - required investigation
- ☐ Vexatious legal complaint - complaint dismissed without investigation
- ☐ Vexatious legal complaint - complaint required investigation
- ☐ None of the above

*** 15. If you have experienced a vexatious complaint, did you feel supported by your institution or professional body? (Select one option)**

- ○ Highly supported
- ○ Supported
- ○ Neutral
- ○ Not well supported
- ○ Entirely unsupported
- ○ Not applicable

*** 16. If you have experienced abusive or diminishing comments, in which fora are these typically delivered or shared? (check all that apply)**

- ☐ E-mail
- ☐ Post
- ☐ Phone calls
- ☐ Texts
- ☐ Twitter
- ☐ Facebook
- ☐ Instagram
- ☐ Youtube
- ☐ Other social media
- ☐ Blogs
- ☐ Websites
- ☐ Public television
- ☐ Public radio
- ☐ Not applicable
- ☐ Other (Please specify) _______________

*** 17. Have you ever had to take legal action or consult law-enforcement officials regarding malicious communications, threats, or claims? (Select one option)**

- ○ Yes
- ○ No
- ○ Not applicable
- ○ Prefer not to say

*** 18. Have negative reactions to public engagement ever caused you mental health problems (depression, anxiety, stress, etc) or otherwise impeded your functioning? (Select one option)**

- ○ Yes - severely
- ○ Yes - considerably
- ○ Yes - to a minor degree
- ○ Unsure
- ○ No
- ○ Prefer not to say

19. Please expand on noteworthy negative situations you have encountered while engaging in health outreach. This is entirely optional, but helps us identify common experiences and pitfalls.

*** 20. Finally, which of the following changes (if any) do you feel would benefit those communicating health the most? (check all that apply)**

- ☐ Increased support from professional bodies
- ☐ Increased support from academic institutions
- ☐ Increased support from advocacy / healthcare bodies
- ☐ Clearer guidelines and better training on public engagement
- ☐ Improved emotional support services
- ☐ Other (Please specify) _______________
- ☐ Practical legal advice
- ☐ More proactive stances from institutions and bodies
- ☐ Dedicated funding for outreach
- ☐ Better protective mechanisms from internet / social media companies
- ☐ None of the above

For any queries, please contact Dr. David Robert Grimes at d.r.grimes@qub.ac.uk or davidrobert.grimes@oncology.ox.ac.uk

Experiences in public communication of medical science & health

Thank you for taking part in this survey. Results will be treated anonymously, but if you are happy for us to contact you and perhaps quote from your experiences if appropriate, please indicate this below.

21. If you would like to be kept up to date on results and follow-up, please enter your e-mail address below.

____

22. We'd be grateful if you'd also supply your twitter handle. This will not be shared without your consent, and is entirely optional, but may prove useful in future work estimating network reach of health information.

____

For any queries, please contact Dr. David Robert Grimes at d.r.grimes@qub.ac.uk or davidrobert.grimes@oncology.ox.ac.uk
