# Supplementary material for: Establishing a taxonomy of potential hazards associated with communicating medical science in the age of disinformation
Source: BMJ Open. 2020 Jul 5;10(7):e035626. doi: 10.1136/bmjopen-2019-035626 (PMC7342820; doi:10.1136/bmjopen-2019-035626)
Supplement: Author's manuscript [file bmjopen-2019-035626.draft_revisions.pdf]

BMJ Open

BMJ Open is committed to open peer review. As part of this commitment we make the peer review history of every article we publish publicly available.

When an article is published we post the peer reviewers' comments and the authors' responses online. We also post the versions of the paper that were used during peer review. These are the versions that the peer review comments apply to.

The versions of the paper that follow are the versions that were submitted during the peer review process. They are not the versions of record or the final published versions. They should not be cited or distributed as the published version of this manuscript.

BMJ Open is an open access journal and the full, final, typeset and author-corrected version of record of the manuscript is available on our site with no access controls, subscription charges or pay-per-view fees (http://bmjopen.bmj.com).

If you have any questions on BMJ Open's open peer review process please email info.bmjopen@bmj.com

BMJ Open

Adverse events - Hazards of communicating medical science in the age of disinformation

Journal:	*BMJ Open*
Manuscript ID	bmjopen-2019-035626
Article Type:	Original research
Date Submitted by the Author:	08-Nov-2019

Complete List of Authors:	Grimes, David; University of Oxford, & Queens University Belfast<br>O'Connor, Robert; Irish Cancer Society

Keywords:	PUBLIC HEALTH, INFECTIOUS DISEASES, ONCOLOGY, MEDICAL JOURNALISM, MEDICAL EDUCATION & TRAINING

SCHOLARONE™
Manuscripts

I, the Submitting Author has the right to grant and does grant on behalf of all authors of the Work (as defined in the below author licence), an exclusive licence and/or a non-exclusive licence for contributions from authors who are: i) UK Crown employees; ii) where BMJ has agreed a CC-BY licence shall apply, and/or iii) in accordance with the terms applicable for US Federal Government officers or employees acting as part of their official duties; on a worldwide, perpetual, irrevocable, royalty-free basis to BMJ Publishing Group Ltd ("BMJ") its licensees and where the relevant Journal is co-owned by BMJ to the co-owners of the Journal, to publish the Work in this journal and any other BMJ products and to exploit all rights, as set out in our licence.

The Submitting Author accepts and understands that any supply made under these terms is made by BMJ to the Submitting Author unless you are acting as an employee on behalf of your employer or a postgraduate student of an affiliated institution which is paying any applicable article publishing charge ("APC") for Open Access articles. Where the Submitting Author wishes to make the Work available on an Open Access basis (and intends to pay the relevant APC), the terms of reuse of such Open Access shall be governed by a Creative Commons licence – details of these licences and which Creative Commons licence will apply to this Work are set out in our licence referred to above.

Other than as permitted in any relevant BMJ Author's Self Archiving Policies, I confirm this Work has not been accepted for publication elsewhere, is not being considered for publication elsewhere and does not duplicate material already published. I confirm all authors consent to publication of this Work and authorise the granting of this licence.

For peer review only

Adverse events - Hazards of communicating medical science in the age of disinformation

David Robert Grimes[1,2*] and Robert O'Connor[3]

1. School of physical sciences, Dublin City University
2. Department of Oncology, University of Oxford
3. Irish Cancer Society, Dublin

Correspondence: davidrobert.grimes@dcu.ie / davidrobert.grimes@oncology.ox.ac.uk

Abstract

Objectives: Disinformation on medical matters has become an increasing public health concern. Public engagement by scientists, clinicians, and patient advocates can contribute towards public understanding of medicine. However, depth of feeling on many issues (notably vaccination and cancer) can lead to adverse reactions for those communicating medical science, including vexatious interactions and targeted campaigns. Our objective in this work is to establish a taxonomy of common negative experiences encountered by those communicating medical science, and suggest guidelines so that they may be circumvented.

Design: We establish a taxonomy of the common negative experiences reported by those communicating medical science, informed by surveying medical science communicators with public platforms.

Participants: 142 prominent medical science communicators (defined as having >1000 Twitter followers and experience communicating medical science on social and traditional media platforms) were invited to take part in a survey, with 101 responses.

Results: 101 responses were analysed. Most participants experienced abusive behaviour (91.9%), including persistent harassment (69.3%) and physical violence and intimidation (5.9%). A substantial number (38.6%) received vexatious complaints to their employers, professional bodies, or legal intimidation. The majority (62.4%) reported negative mental health sequelae due to public outreach, including depression, anxiety, and stress. A significant minority (19.8%) were obligated to seek police advice or legal counsel due to actions associated with their outreach work. While the majority targeted with vexatious complaints felt supported by their employer / professional body, 32.4% reported neutral, poor or non-existent support.

Conclusions: Those engaging in public outreach of medical science are vulnerable to negative repercussions, and we suggest guidelines for professional bodies and organisations to remedy some of these impacts on front-line members.

Strengths and limitations

- Establishes a taxonomy of adverse reactions those communicating medical science can encounter.
- Suggests guidelines for professional bodies, research institutes, and medical centres to circumvent the worst impacts on members engaged in public outreach on medical science.
- The taxonomy of adverse impacts was informed by surveying prominent communicators of medical science.
- Survey results should only be taken as indicative of the scope of the issue at this juncture.
- Much further research is needed to ascertain how the medical community can best act to counter the rise of medical disinformation whilst protecting practitioners.

For peer review only

Introduction

Despite being fundamental to societal well-being, many aspects of medical science remain poorly understood and frequently distrusted. Disinformation undermining health science and evidence-based medicine has increased markedly in the era of social media, and dangerous misconceptions abound, from perceived cancer risks and ostensible cures [1] to dangerous falsehoods about vaccination [2]. Improving public awareness and understanding of science and medicine is imperative if we are to maintain continued progress in research endeav-ours, and scientists, physicians, and science communicators have a crucial role to play in shaping public perceptions. Medical science is largely publicly funded, and direct communication of research with the wider public can be extraordinarily beneficial on a societal level. Accordingly, public engagement has become a prerequisite for many funding bodies. Informed engagement by patient advocates and media gures too can have marked impact on public understanding of medicine, empowering the public with facts with which to make important health decisions.

Improving public understanding of medical science is vital, as there many scenarios where public perception (or a vocal subset of that) is starkly at odds with scientific consensus. Frequently, medical science contradicts a narrative strongly held by particular groups within the wider public. For our purposes, we define a ‘narrative’ as a world-view or mindset shared by a particular subgroup, which unifies that grouping. Narratives are often articles of faith, empowerment or comfort, frequently unsupported by available evidence or at odds with scientific consensus. For clarity, we concentrate herein on situations where there is no reputable evidence for a narrative, or where overwhelming scientific consensus is firmly against that viewpoint.

Misguided narratives can be supremely damaging, and the anti-vaccine movement is perhaps the most obvious example of this. Despite their life-saving efficacy of vaccination, opposition has existed since the time of Jenner [3]. The rise of social media has seen significant propagation of anti-vaccine narratives [4, 5, 6], driving uptake rates down and causing serious harm worldwide [7, 8]. In 2018, Europe saw the highest number of cases of measles cases in 20 years, numbering over 82,525 cases with at least 72 death - over fifteen-fold the figures from 2016 [9]. Such is the extent of the problem that in 2019 the World Health Organisation described vaccine hesitancy as a “Top ten threat to global health" [10]. Exposure to anti-vaccine conspiracy to anti-vaccine conspiracy theory is a leading factor in parental intention to vaccinate [8], and evidence to dates suggests that the deluge of vaccine disinformation across social media is extremely damaging to public understanding and health.

Other strongly-held narratives which clash with the weight of available scientific evidence include the claims propounded by the anti-fluoride movement [11, 12], the beliefs of the electromagnetic hypersensitivity movement [13], and the narratives of alternative medicine [14, 15]. Cancer patients are especially vulnerable to misinformation, and frequently targeted by charlatans and the misguided [16]. Consequences of this can be severe, with patients sometimes delaying or refusing conventional treatment. The net result of this is diminished survival statistics for those who engage with cancer pseudoscience, due to delayed treatment and sometimes rejection of conventional medicine. In some instances, subscribing to un-proven or disproven modalities could approximately half survival time [17].

For peer review only

While health falsehoods has always existed, the social media age has created new avenues for misinformation (misinformed advice) and disinformation (deliberate falsehoods) to propagate [16], rapidly bringing discredited ideas and dangerous pseudoscience to vast new audiences. Scaremongering stories from dubious outlets propagate more readily than reliable fact-based information from reputable sources [18, 19]. In 2016, over half of all cancer stories shared on Facebook were medically unsound. Some have harnessed pseudoscience to sell questionable diets, supplements, and books, to the detriment of public understanding. Internet health gure Joseph Mercola, for example, made over $7 million in 2010 alone, proffering highly dubious treatments and advice [20], including denigration of conventional therapies for cancer. Mercola is far from unique, and such proclamations have huge potential for patient harm.

To counter this, public outreach by scientists, physicians, and evidence-based health advocates must be a crucial element to counter damaging ctions, and strive for evidence-based medicine. A physician's recommendation, for example, is central to parental decisions to vaccinate [21]. Addressing patient concerns improves public health, and personal engagement by researchers and physicians can have a positive impact on public perception. Patient advocates and media gures have substantial ability to shift public perception; after Ireland saw HPV vaccine uptake drop from 87% to 51%, an alliance of healthcare professionals, researchers, and patient advocates were instrumental in countering the dominant falsehoods, and Ireland has seen a dramatic recovery in vaccine uptake rates [22]. To make inroads against the deluge of dubious health claims to which we're subjected, it's vital that scientists, clinicians, and patient groups must be on the vanguard of efforts to counter-act misinformation.

Those engaging in public outreach, however, often encounter enmity for publicly advocating scientific evidence. Scientific consensus often runs contrary to deeply-held beliefs, leading to certain groups attempting to undermine legitimate scientific communication. Motivations for this are multi-faceted, often depending on very specific circumstances. Conspiratorial thinking underpins many narratives, and those attempting to communication science are often vilified as “shills", or agents of a nebulous “Big Pharma". The phenomenon of identity protective cognition is also commonly encountered [23] and narrative believers frequently attacking those who cast doubt on their beliefs. Even when handled with sympathy and compassion, professional and patient advocates who challenge misconceptions can become targets for certain individuals and groups.

These negative responses can range from verbal abuse to coordinated harassment campaigns, and even violence. Aside from being deeply unsettling, such responses can cause professional and personal problems for those targeted. With the increasing emphasis on public engagement and ubiquity of the internet, this subject warrants urgent consideration, as there are currently few clear guidelines for researchers, clinicians, or patient advocates engaging in outreach work. Nor indeed is there a unified understanding of how adverse effects can manifest, and institutions and professional bodies are typically ill-equipped or muted in their support. This leaves those in the public eye or studying contentious topics vulnerable to vexatious complaints and even physical harm. Without awareness of this reality, a less than ideal situation where professional bodies can be weaponized against researchers can too easily emerge. Accordingly, the aim of this work is to ascertain the typical experiences of those communicating medical science and identify how these might be counteracted.

For peer review only

Figure 1 A non-exhaustive taxonomy of negative experiences encountered by individuals engaging in public communication of health science. Subsections discussed in text

.

Methods

Non-exhaustive taxonomy of adversarial tactics

An non-exhaustive taxonomy was constructed from interviews on the experiences of those communicating medical science to the public. While non-exhaustive, it forms a useful basis for more systematic investigation. Adversarial experiences in communicating medicine were broadly strati ed into ve distinct classes, as illustrated graphically in figure 1. These sub-types are detailed in detail in table 1.

For peer review only

Table 1 A non-exhaustive taxonomy of common adversarial tactics

**Misrepresentation of scientific evidence / expertise**		
Straw-manning	Misrepresenting scientific arguments to make them amenable to ridicule or attack, and to deflect or obscure evidence that undermines a particular narrative.	*"Mercury is toxic, yet scientists put it in vaccines!"* - This statement belies importance of dose and ignores the fact there is no evidence for harm from thimerosal in vaccines.
Cherry-picking / Quote-mining	Selective, manipulative filtering of scientific evidence or expert statements, taken out of context and employed to undermine the evidence-base or promote a narrative.	*"THC kills cancer, but doctors don't want you to know about cannabis cures."* - THC can kill cells in a petri dish, but killing plated cells is entirely different from treating human cancer.
Shifting the burden of proof	Insisting the onus is on scientists to 'disprove' claims rather than offering any evidence or rationale for assertions made.	*"GMOs are toxic, and scientists should prove us wrong*" - This assertion is untrue, and onus lies on those making the claim to proffer evidence for it.
**Discreditation attempts**		
Questioning qualification	Casting doubt on one's ability to question claims at hand. Typically, this ostensible scepticism is not extended to purported experts sharing claims supportive of the narrative.	*"This patient advocate isn't an expert, so they can't say this vaccine is safe!* " - One does not need to be an expert immunologist in this case to accurately reflect the medical consensus on vaccination.
Alleging vested interests	Claims that the speaker is compromised due to some apparent conflict of interest or that experts are otherwise lacking impartiality.	*"Who's paying you to say this?"* - Unsubstantiated allegation to deflect from absence of evidence for a narrative or claim.
Asserting Conspiracy theory	Allegations that the scientist, physician, or patient advocate is part of some conspiracy to suppress the truth or spread false information, either as a pawn or an active player.	*"She's part of a pharma cover-up to suppress natural cancer cures!"* - Appeals to conspiracy theory function to distract from lack of evidence.
**Dubious Amplification of pseudo-scientific narratives**		
Media Targeting (by-passing the gatekeepers	Targeting traditional or online media outlets to amplify dubious narratives, typically by-passing traditional gatekeepers (science / health journalists etc) who would otherwise be more likely to spot pseudoscience.	Pitching dubious health claims to journalists as human interest stories - This approach was successfully used by anti-vaccine activists to push the dis credited link between autism and the MMR vaccine between 1998-2000.
Astroturfing / sock-puppeting	Use of fake social media accounts / fictitious pressure groups to provide an illusion of grass-roots support for a particular narrative	Example: Accounts which spring up once an initial anti-fact site is blocked but which include misinformation consistent with the originator's initial social media accounts.
Celebrity endorsement	Celebrities and influencers can have disproportionately large impact on the perception of public even in areas where they have no relevant expertise or knowledge.	There are numerous examples of this, especially in relation to anti-vaccine activism, including actors and models being cited for their purported knowledge of complex health issues.
**Malicious complaints / abuse of regulatory frameworks**		
Poisoning the well / Smear campaigns	The spreading of malicious claims regarding an individual's professional or personal conduct to undermine them or discourage others from engaging with them.	*"I've heard that doctor abuses patients"* - Inflammatory slurs such as these are designed to discredit, and are not in any way substantiated, but calculated to invoke disgust or contempt.
Vexatious complaints to employers	Making calculated complaints to one's employer or threatening to do so in order to intimidate them into silence.	Exaggerated / misleading accounts of interactions with public advocates and demands to censure them, typically aimed at an individual's university or employer.
Vexatious complaints to regulatory bodies	Abusing procedures of professional bodies to target a researcher / medic who presents a challenge to a narrative. These may also include unwarranted freedom of information requests or vexatious parliamentary questions.	Registering complaints with a medical regulatory body against a doctor for their advocacy of evidence-based positions. Physicians especially vulnerable, as typically all complaints must be investigated, regardless of merit.
**Interpersonal harassment and intimidation**		
Harassment / abuse	Harassment can take many forms, and personal abuse is perhaps most common. Threats (implied and direct) are often employed.	Abusive language made publicly or in direct messages, and posting of private contact details, phone numbers, addresses etc.
Legal threats	Legal notices and mechanisms can also be utilised to silence researchers questioning a narrative, from cease and desist notices to defamation claims.	Threatening to bring an advocate to court for alleged defamation, also used judiciously to limit independent investigation on pseudoscientific narratives
Physical intimidation	Implicit or explicit threats of physical violence is an Unfortunately potent method of intimidating scientists into silence. This includes threats of physical violence or rape (the latter usually directed at female discussants)	Stalking of private abodes, explicit threats, or actions like spitting etc. There are instances of security being required for scientific meetings on publicly contentious subjects, due to implications of or threats of violence.

For peer review only

Table 2 Survey participants

Variable	Participants
Invited to partake<br>Total completed	N=142<br>N=101
Gender	Female (N=55)<br>Male (N = 44)<br>Non-Binary / Undisclosed (N=2)
Affiliation	University / Medical Centre (N = 52)<br>Una liated (N = 26)<br>Media organisation (N = 20)<br>Charity (N = 11)<br>Political organisation (N=4)
Profession	Medical professional (N = 23)<br>Scientist / Researcher (N = 20)<br>Science communications (N = 18)<br>Patient Advocate (N = 16)<br>Health Policy (N=5)
Years involved	Average 10.7 Years (Range 2 - 30)

Survey of negative experiences

142 people were invited to undertake a brief survey aimed at capturing what if any negative experiences they might have suffered whilst advocating evidence-based medical information in public forums, with 101 (71.1%) responding. Selection criteria was based on having more than 1000 twitter followers, predominantly focused on English-speaking public communica-tion of health science and with media experience in both traditional and online channels. This survey was undertaken for indicative purposes, with participants invited from a variety of backgrounds and subject areas. Data was collected from a non-randomised group with no expectation of transferable findings, and was thus exempt from requiring HRA / HRB approval. Participant details are given in table 2. Topics covered / channels of engagement are depicted in figure 2(a).

Patient and Public Involvement

As patient advocates play a substantial role to play in combatting misinformation on medical issues, several who met the inclusion criteria were invited to take part, with 15.8% of respondents being patient advocates.

For peer review only

Results

Topics covered, channels of engagement, and mediums for negative encounters are shown in figure 2. Twitter is disproportionately represented, as prominence on that platform was part of the selection criteria. Other fora cited included books, documentaries, newspapers, podcasts - and in one instance criticism under parliamentary privilege. A majority (N = 70, 69.3%) had experienced targeted ire from at least one grouping. The most common groupings for negative reactions were anti-vaccine and alternative medicine groups respectively (N = 43 each, 42.6%) followed by dietary advocates (N = 26, 25.7%), 'wellness' groups (N = 17, 16.8%), religious and chronic illness groups (both N = 15, 14.9%), anti- fluoride and autism focused groups (both N =12, 11.9%). Others cited by 3 or less respondents included electromagnetic hypersensitivity factions, conspiracy theorists and anti-GMO organisations.

Figure 3 depicts types of experiences reported by participants, ranging from the relatively minor to the severe. Of participants surveyed, a majority (N = 63, 62.4%) reported negative fallout from public engagement had caused them at least some negative mental health sequelae, including depression, anxiety, and stress. Most of this was reported as minor, but considerable or significant mental health ramifications were reported by 15 respondents. Of the participants, 20 (19.8%) were obligated to seek police advice / legal counsel as a direct result of targeted actions associated with their outreach work. Of those receiving vexatious complaints (N = 39, 38.6% of all respondents), most (67.6%) felt supported or well supported by their institution, employer, or professional body, with whilst 16.2% deemed support to be neutral, with an equal number (16.2%) feeling poorly or entirely unsupported. Predictably perhaps, gender specific abuse was far more likely to be directed at women (40% of female respondents) than men (6.8% of male respondents), with this difference being highly significant ($p << 0.001$, calculated by Welch's t-test)

Figure 2 (a) Topics covered by participants (b) channels of engagement for subjects surveyed (c) Fora for negative interactions

Figure 3 Proportion of negative experiences recorded including (a) abuse experienced (b) violence and intimidation (c) vexatious complaints.

For peer review only

Discussion

In a globalised information age, medical science can appear disconnected and aloof from those it serves to help. Educational and professional bodies (including universities and medical centres) have a unique societal role to inform their peers and public on evidence-based medicine, and a responsibility to adjust to modern communications realities. We can collectively no longer remain on the fence in supporting health information advocacy. While being mindful not to over-infer from our survey, we can use it as a basis to make some suggestions. It is vital to have proactive strategies in place to support those engaging in medical outreach, and to maintain a high calibre for public discussion. It is also crucial that those engaging in outreach are cognizant of the potential pitfalls, and afforded ample support.

Suggested guidelines for professional bodies and employers

While by no means comprehensive, the following guidelines might be bene cial in establish-ing policy for dealing with issues that can arise.

1 Educational / professional organisations must recognise a commitment to support evidence-based actions by their members. This may require oversight of such activities and investment in the governance / training resources to protect members willing to act as advocates.

2 Institutions and professional bodies should have robust measures in place to oversee communication activities associated with their members, and to make assessments as to whether individuals are communicating established facts in good faith or are contributing to undermining of fact with potential legal / reputational damage.

3 When institutions receive complaints regarding members, the subject must be a orded presumption of innocence rather than being served with reactionary and inflexible procedure, lest the institution might become an unwilling tool against science.

4 In case of disputes, competent and impartial individuals should be engaged to independently assess complaints, cognisant of vital background and context.

5 Coordination between press offices and those engaged in outreach would improve communication, preemptively identifying those likely to be targets for malicious tactics.

6 Support for those identified as being on engagement front-lines should be maintained, with clear legal advice / institutional support for targeted members.

7 Organisations must be vocal in supporting public-facing members, willing to issue strong rebuttals of vexatious complaints against individuals.

8 Professional bodies and employers should promote strive to promote both scientific freedom of speech, and strive to champion evidence-based advocacy.

9 When possible, those expected to engage in outreach should be trained in methods that reduce opportunities for personal attacks.

Conclusions

The question of how we best communicate health science in the modern era is an area where more research is urgently required, especially on the role of social media, and optimum ways physicians, researchers, and other public-facing figures can promote good medical science and mitigate falsehoods. The suggestions herein ought to be taken as a starting point, with discussion evolving as improved evidence materialises. There are wider problems implicit in all this those communicating science cannot tackle in isolation; social media regulation

For peer review only

particularly is a serious issue, both in regards to the spreading of misinformation / disinformation, and with respect to procedures preventing the weaponisation of social media platforms. Social media platforms must ultimately be made answerable to regulatory oversight, just as every other important aspect of life is; claims of innocence are unconvincing when their business model is so clearly dependent on advertising engagement at the cost of lives. The problem of poor reporting and false balance [24] in conventional media outlets also must be considered, and there is significant scope for scientists and doctors to contribute to policy in these areas. There is ample evidence that physicians and scientists have an important role to play in combatting health disinformation, as has recently been argued by the authors in relation to vaccination for the British Medical Journal [25]. But equally, it is crucial that those engaging in this vital work have the requisite support from their institutions, so that deleterious consequences of laudable outreach work might be circumvented. It is increasingly clear that disinformation about medicine and illness has become ubiquitous, with severe consequence for both our collective health and public understanding of medical science. Scientists and physicians must be at the vanguard of the pushback against these dangerous falsehoods – our societal well-being depends upon it.

Electronic Supplementary Material

A full version of the questionnaire and further analysis can be found in the electronic supplementary material.

Acknowledgements
The authors would like to thank the scientists, physicians, and patient advocates who gave their time to discuss the issues they face in communicating science, and for sharing their insights. In particular, the authors would like to profoundly thank Dr. Laura J Brennan for her invaluable input; Laura was present and helped conceive the concept from which this paper sprung. She was a passionate advocate evidence-based science communication and especially for the HPV vaccine, championing it despite enduring a life-limiting and difficult cervical cancer diagnosis. Following a con dence crisis in the vaccine in Ireland driven by anti-vaccine activism, her tireless work was a substantial factor in reversing the damage wrought by disinformation. Laura passed away in March 2019, but while we have lost a friend and colleague, hers is a legacy that will resonate for generations. Laura embodied precisely how vital health communication is to promoting public health; Ireland, and the world, owe her a huge debt, and we dedicate this work to her memory.

Contributor statement
All authors contributed equally to this work.

Competing interests
The authors declare that they have no competing interests.

Funding statement
The authors declare that they have no specific funding for this work.

Data Sharing
Full survey forms available in electronic supplementary material.

References

1. Shahab, L., McGowan, J.A., Waller, J., Smith, S.G.: Prevalence of beliefs about actual and mythical causes of cancer and their association with socio-demographic and health-related characteristics: Findings from a cross-sectional survey in england. European Journal of Cancer 103, 308{316 (2018)
2. Kata, A.: A postmodern pandora's box: anti-vaccination misinformation on the internet. Vaccine 28(7), 1709{1716 (2010)
3. Poland, G.A., Jacobson, R.M.: The age-old struggle against the antivaccinationists. New England Journal of Medicine 364(2), 97{99 (2011)
4. Zimmerman, R.K., Wolfe, R.M., Fox, D.E., Fox, J.R., Nowalk, M.P., Troy, J.A., Sharp, L.K.: Vaccine criticism on the world wide web. Journal of medical internet research 7(2), 17 (2005)
5. Kata, A.: Anti-vaccine activists, web 2.0, and the postmodern paradigm{an overview of tactics and tropes used online by the anti-vaccination movement. Vaccine 30(25), 3778{3789 (2012)
6. Grimes, D.R.: On the viability of conspiratorial beliefs. PloS one 11(1), 0147905 (2016)
7. Goertzel, T.: Conspiracy theories in science. EMBO reports 11(7), 493{499 (2010)
8. Jolley, D., Douglas, K.M.: The e ects of anti-vaccine conspiracy theories on vaccination intentions. PloS one 9(2), 89177 (2014)
9. Thornton, J.: Measles cases in Europe tripled from 2017 to 2018. British Medical Journal Publishing Group (2019)
10. Organization, W.H., et al.: Ten threats to global health in 2019. 2019. Retrieved January (2019)

For peer review only

For peer review only - http://bmjopen.bmj.com/site/about/guidelines.xhtml

11. Freeze, R.A., Lehr, J.H.: The Fluoride Wars: How a Modest Public Health Measure Became America's Longest-Running Political Melodrama. John Wiley & Sons, ??? (2009)
12. Grimes, D.R.: Commentary on “are fluoride levels in drinking water associated with hypothyroidism prevalence in england? a large observational study of gp practice data and fluoride levels in drinking water". J Epidemiol Community Health 69(7), 616{616 (2015)
13. Grimes, D.R., Bishop, D.V.: Distinguishing polemic from commentary in science: Some guidelines illustrated with the case of sage and burgio (2017). Child development 89(1), 141{147 (2018)
14. Singh, S., Ernst, E.: Trick or Treatment: The Undeniable Facts About Alternative Medicine. WW Norton & Company, (2008)
15. Grimes, D.R.: Proposed mechanisms for homeopathy are physically impossible. Focus on Alternative and Complementary Therapies 17(3), 149{155 (2012)
16. The, L.O.: Oncology," fake" news, and legal liability. The Lancet. Oncology 19(9), 1135 (2018)
17. Johnson, S.B., Park, H.S., Gross, C.P., James, B.Y.: Complementary medicine, refusal of conventional cancer therapy, and survival among patients with curable cancers. JAMA oncology 4(10), 1375{1381 (2018)
18. Vosoughi, S., Roy, D., Aral, S.: The spread of true and false news online. Science 359(6380), 1146{1151 (2018)
19. Brady, W.J., Wills, J.A., Jost, J.T., Tucker, J.A., Van Bavel, J.J.: Emotion shapes the diffusion of moralized content in social networks. Proceedings of the National Academy of Sciences 114(28), 7313{7318 (2017)
20. Smith, B.: Dr. mercola: Visionary or quack? Chicago (2012)
21. Rosenthal, S., Weiss, T.W., Zimet, G.D., Ma, L., Good, M., Vichnin, M.: Predictors of hpv vaccine uptake among women aged 19{26: importance of a physician's recommendation. Vaccine 29(5), 890{895 (2011)
22. Corcoran, B., Clarke, A., Barrett, T.: Rapid response to hpv vaccination crisis in ireland. The Lancet 391(10135), 2103 (2018)
23. Kahan, D.M.: Misconceptions, misinformation, and the logic of identity-protective cognition (2017)
24. Grimes, D.R.: A dangerous balancing act. EMBO reports (2019)
25. Grimes, D.R.: “David Robert Grimes: Vaccines-How Can We Counter Misinformation Online?” The BMJ, 7 Nov. 2019, blogs.bmj.com/bmj/2019/11/06/david-robert-grimes-vaccines-how-can-we-counter-misinformation-online/.

For peer review only

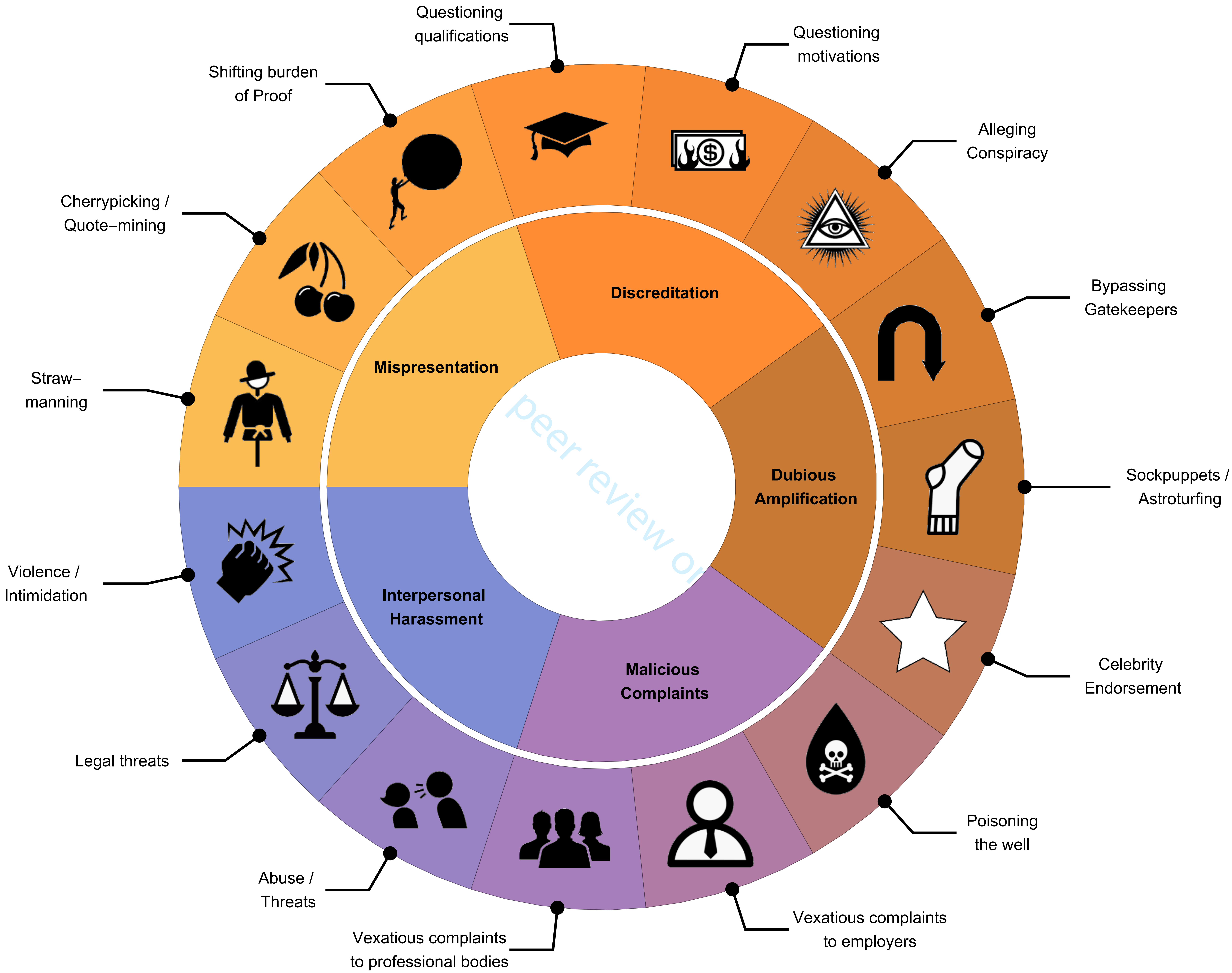
Discreditation
Dubious Amplification
Malicious Complaints
Interpersonal Harassment
Mispresentation
Questioning qualifications
Questioning motivations
Alleging Conspiracy
Bypassing Gatekeepers
Sockpuppets / Astroturfing
Celebrity Endorsement
Poisoning the well
Vexatious complaints to employers
Vexatious complaints to professional bodies
Abuse / Threats
Legal threats
Violence / Intimidation
Straw–manning
Cherrypicking / Quote–mining
Shifting burden of Proof

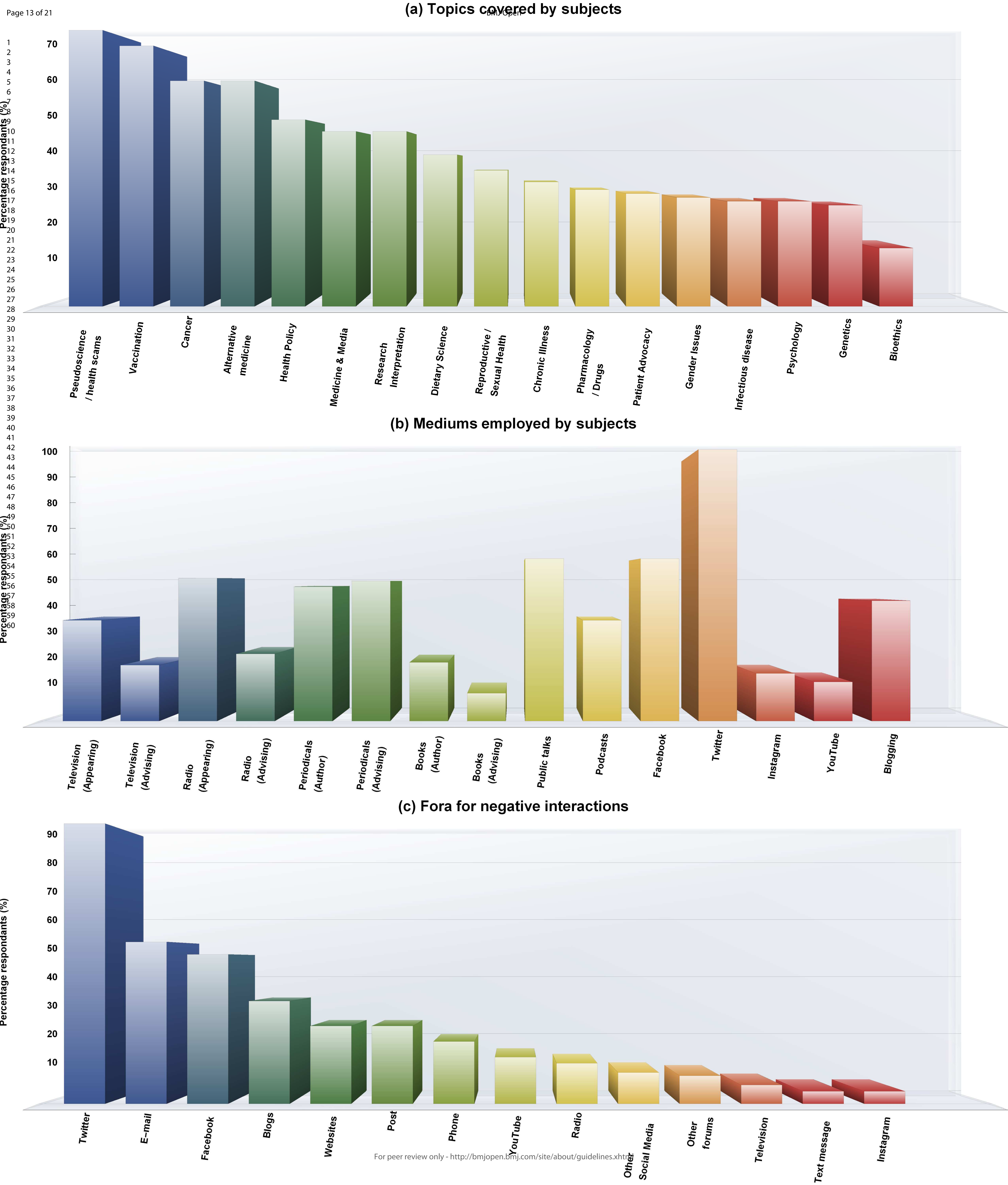
(a) Topics covered by subjects
Percentage respondants (%)
10
20
30
40
50
60
70
Pseudoscience / health scams
Vaccination
Cancer
Alternative medicine
Health Policy
Medicine & Media
Research Interpretation
Dietary Science
Reproductive / Sexual Health
Chronic Illness
Pharmacology / Drugs
Patient Advocacy
Gender Issues
Infectious disease
Psychology
Genetics
Bioethics
(b) Mediums employed by subjects
Percentage respondants (%)
10
20
30
40
50
60
70
80
90
100
Television (Appearing)
Television (Advising)
Radio (Appearing)
Radio (Advising)
Periodicals (Author)
Periodicals (Advising)
Books (Author)
Books (Advising)
Public talks
Podcasts
Facebook
Twitter
Instagram
YouTube
Blogging
(c) Fora for negative interactions
Percentage respondants (%)
10
20
30
40
50
60
70
80
90
Twitter
E-mail
Facebook
Blogs
Websites
Post
Phone
YouTube
Radio
Other Social Media
Other forums
Television
Text message
Instagram

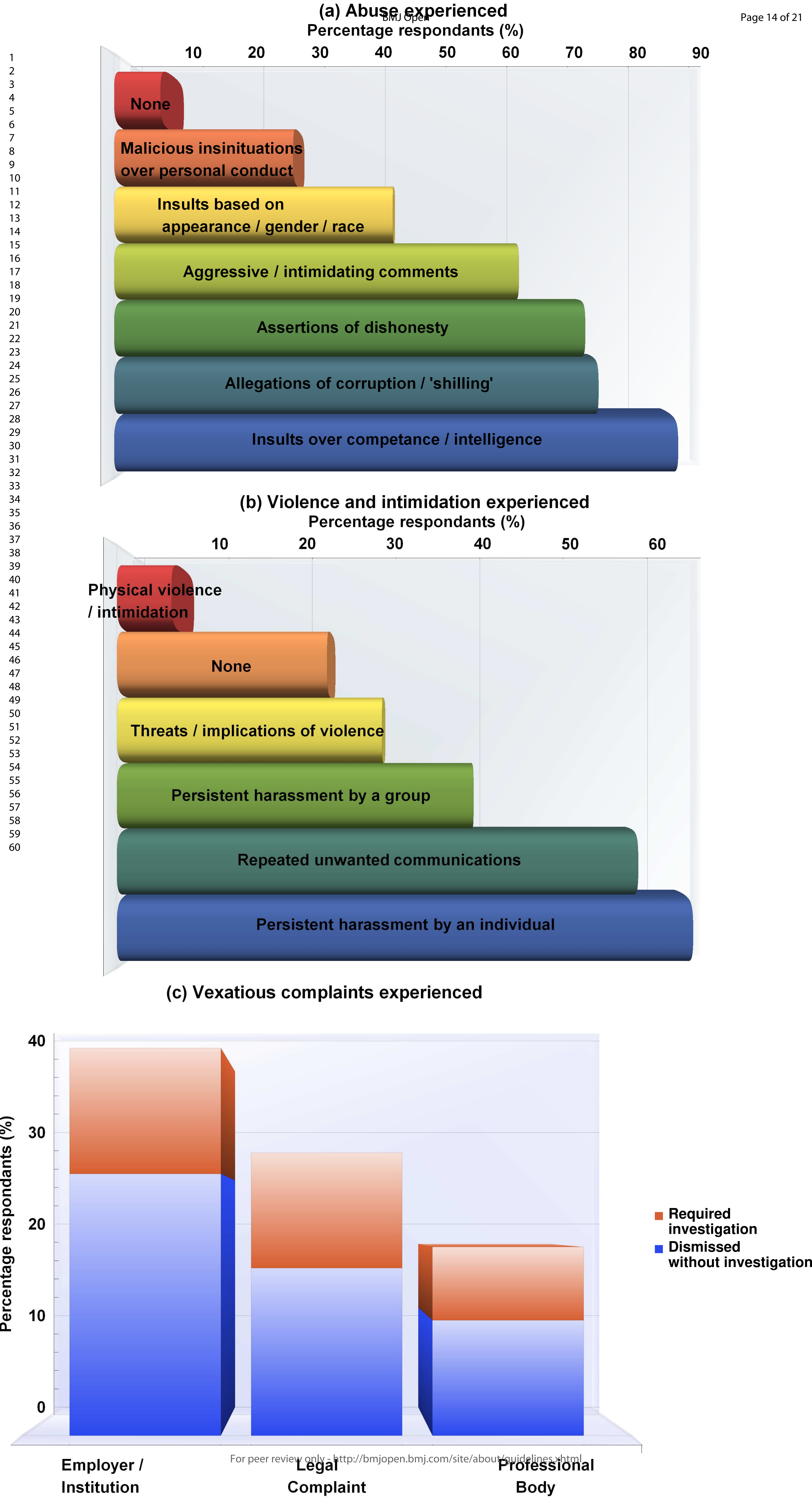
(a) Abuse experienced
Percentage respondants (%)
10 20 30 40 50 60 70 80 90
None
Malicious insinituations over personal conduct
Insults based on appearance / gender / race
Aggressive / intimidating comments
Assertions of dishonesty
Allegations of corruption / 'shilling'
Insults over competance / intelligence
(b) Violence and intimidation experienced
Percentage respondants (%)
10 20 30 40 50 60
Physical violence / intimidation
None
Threats / implications of violence
Persistent harassment by a group
Repeated unwanted communications
Persistent harassment by an individual
(c) Vexatious complaints experienced
Percentage respondants (%)
0 10 20 30 40
Employer / Institution
Legal Complaint
Professional Body
Required investigation
Dismissed without investigation

* Required Information

Experiences in public communication of medical science & health

This survey is part of a research project that aims to better understand the experiences of medical professionals, scientific researchers, patients advocates, health journalists, & policy makers in communicating health issues to the public. It consists of 20 short questions, & should take 5-10 minutes to complete. All responses are anonymous, and their content will be used for research purposes only. If you might be willing to contribute more about your experiences we invite you to inform us of this on the final page of the survey. For any questions, please e-mail d.r.grimes@qub.ac.uk

*** 1. Please select that which best describes your primary role (Select one option)**

- ○ Medical Professional
- ○ Scientific Communications
- ○ Scientist / Researcher
- ○ Health Journalism
- ○ Patient Advocate
- ○ Health Policy
- ○ Other (Please specify) __________

*** 2. Please specify your gender (Select one option)**

- ○ Female
- ○ Non-Binary
- ○ Male
- ○ Prefer not to say

*** 3. Please indicate which of the following organsiations you are professionally affiliated with that are directly relevant to your outreach efforts.**

- ☐ University / research institute
- ☐ Media outlet
- ☐ Medical institute
- ☐ Political grouping
- ☐ NGO / Charity
- ☐ Non-affiliated (Patient Advocate / independent)
- ☐ Other (Please specify) ______________

*** 4. Please select the topic or topics you frequently communicate about with the public. Please note that this includes topics you might debunk or criticize. Select all that apply.**

- ☐ Vaccination
- ☐ Alternative Medicine
- ☐ Medicine in media
- ☐ Dietary Science
- ☐ Pseudoscience / scams
- ☐ Medical science
- ☐ Cancer
- ☐ Chronic illness
- ☐ Bioethics
- ☐ Health Policy
- ☐ Infectious diseases
- ☐ Interpretation of research
- ☐ Reproductive / Sexual Health
- ☐ Pharmacology / drugs
- ☐ Gender issues
- ☐ Patient Advocacy
- ☐ Psychological issues
- ☐ Genetics
- ☐ Other (Please specify) ______________

*** 5. What media do you use for public engagement? Select all that apply.**

- ☐ Television (appearances)
- ☐ Television (advising / quoted)
- ☐ Radio (appearances)
- ☐ Radio (advising / quoted)
- ☐ Newspapers / Magazines (Author)
- ☐ Newspapers / Magazines (advising / quoted)
- ☐ Books (Author)
- ☐ Books (Advising / quoted)
- ☐ Public talks
- ☐ Podcasts
- ☐ Facebook
- ☐ Twitter
- ☐ Instagram
- ☐ Youtube
- ☐ Blogging
- ☐ Other (Please specify) _______________

*** 6. Approximately how long have you been communicating medical issues to the public? Please round to the nearest whole year**
(Enter a value between 0 and 120)

____

*** 7. Please select what best describes the extent of your public engagement (Select one option)**

- ○ I am heavily involved in public outreach
- ○ I am considerably involved in public outreach
- ○ I am involved in public outreach
- ○ I am lightly involved in public outreach
- ○ My vocation is media or outreach centred
- ○ Other (Please specify) ___________

For any queries, please contact Dr. David Robert Grimes at d.r.grimes@qub.ac.uk or davidrobert.grimes@oncology.ox.ac.uk

For peer review only - http://bmjopen.bmj.com/site/about/guidelines.xhtml

Experiences in public communication of medical science & health

Part II: Engagement experiences

8. In your opinion, how frequently do the following sentiments apply to your experiences of public engagement?

	Always	Frequently	Sometimes	Infrequently	Never	Unsure
*(a) I feel my efforts contribute to public understanding	○	○	○	○	○	○
*(b) I find engagement prompts constructive and respectful discussion	○	○	○	○	○	○
*(c) Engagement is mutually informative	○	○	○	○	○	○
*(d) My contributions are welcomed & appreciated	○	○	○	○	○	○
*(e) Engagement increases my personal understanding	○	○	○	○	○	○
*(f) I feel I correct some misconceptions	○	○	○	○	○	○
*(g) I find my engagement changes minds & informs	○	○	○	○	○	○
*(h) I consider outreach personally rewarding	○	○	○	○	○	○
*(i) My efforts feel futile	○	○	○	○	○	○
*(j) Engagement takes a toll on my mental health & well-being	○	○	○	○	○	○
*(k) I find my efforts are taken in good faith by others	○	○	○	○	○	○

*** 9. Please indicate whether you have experienced any of the following negative experiences during your engagement efforts. Select all that apply. Note that for the purposes of this survey, vexatious complaints are defined as those raised chiefly to harass or intimidate.**

- ☐ Personal abuse
- ☐ Personal smears
- ☐ Professional smears
- ☐ Physical threats
- ☐ Threats of sexual violence
- ☐ Harassment from individuals
- ☐ Coordinated harassment from specific groups
- ☐ Questioning of one's motivations
- ☐ Other (Please specify) _______________
- ☐ Sexist or gender-specific abuse
- ☐ Racist of ethnic-specific abuse
- ☐ Intimidation attempts
- ☐ Vexatious legal threats
- ☐ Vexatious complaints to employers
- ☐ Vexatious complaints to professional bodies
- ☐ Persistent unwanted communication
- ☐ No negative experiences

*** 10. How would you best describe your feelings about your outreach experiences to date? (Select one option)**

- ◯ Largely rewarding
- ◯ Rewarding
- ◯ Mixed
- ◯ Not very rewarding
- ◯ Not at all rewarding
- ◯ Other (Please specify) ___________

For any queries, please contact Dr. David Robert Grimes at d.r.grimes@qub.ac.uk or davidrobert.grimes@oncology.ox.ac.uk

For peer review only - http://bmjopen.bmj.com/site/about/guidelines.xhtml

Experiences in public communication of medical science & health

* 11. **If you have been the recipient of abuse or personal / professional smears stemming from your engagement, please indicate which of the following you've experienced (check all that apply)**

- ☐ Aggressive or intimidating comments
- ☐ Insults based on appearance, race, or gender
- ☐ Malicious comments about one's motivations / allegations of 'corruption' or 'shilling'
- ☐ Malacious comments about one's personal or sexual conduct
- ☐ Other (Please specify) _______________
- ☐ Assertions that you are dishonest or deliberately misleading
- ☐ Insults about your professional competence or intelligence
- ☐ I have not experienced any of the above

* 12. **Please indicate whether you have been subjected to any of the following (check all that apply)**

- ☐ Threats or implications of physical violence
- ☐ Physical violence or intimidation
- ☐ Spreading of malicious rumours
- ☐ Repeated unwanted communications
- ☐ Other (Please specify) _______________
- ☐ Persistent trolling / harassment by an individual
- ☐ Persistent trolling / harassment by a group
- ☐ None of the above

* 13. **If you have been the victim of targeted abuse for your outreach work by a group or community, what best describes that grouping? Choose all that apply if relevant**

- ☐ Anti-vaccine groups
- ☐ Dietary advocates
- ☐ Chronic illness groups
- ☐ Anti-fluoride groups
- ☐ Electromagnetic hypersensitivity groups
- ☐ Other (Please elaborate as appropriate) _______________
- ☐ Religious groupings
- ☐ Alternative medicine advocates
- ☐ "Wellness" groups
- ☐ Autism-focused groups
- ☐ I have not encountered this

* 14. **Please indicate whether you have experienced any of the following. Note that for the purposes of this survey, vexatious complaints are defined as those raised chiefly to harass or intimidate.**

- ☐ Vexatious complaint to employer or institution - complaint dismissed without investigation
- ☐ Vexatious complaint to employer or institution - required investigation
- ☐ Vexatious complaint to a professional body - complaint dismissed without investigation
- ☐ Vexatious complaint to a professional body - required investigation
- ☐ Vexatious legal complaint - complaint dismissed without investigation
- ☐ Vexatious legal complaint - complaint required investigation
- ☐ None of the above

*** 15. If you have experienced a vexatious complaint, did you feel supported by your institution or professional body? (Select one option)**

- ○ Highly supported
- ○ Supported
- ○ Neutral
- ○ Not well supported
- ○ Entirely unsupported
- ○ Not applicable

*** 16. If you have experienced abusive or diminishing comments, in which fora are these typically delivered or shared? (check all that apply)**

- ☐ E-mail
- ☐ Post
- ☐ Phone calls
- ☐ Texts
- ☐ Twitter
- ☐ Facebook
- ☐ Instagram
- ☐ Other (Please specify) _______________
- ☐ Youtube
- ☐ Other social media
- ☐ Blogs
- ☐ Websites
- ☐ Public television
- ☐ Public radio
- ☐ Not applicable

*** 17. Have you ever had to take legal action or consult law-enforcement officials regarding malicious communications, threats, or claims? (Select one option)**

- ○ Yes
- ○ No
- ○ Not applicable
- ○ Prefer not to say

*** 18. Have negative reactions to public engagement ever caused you mental health problems (depression, anxiety, stress, etc) or otherwise impeded your functioning? (Select one option)**

- ○ Yes - severely
- ○ Yes - considerably
- ○ Yes - to a minor degree
- ○ Unsure
- ○ No
- ○ Prefer not to say

only

19. **Please expand on noteworthy negative situations you have encountered while engaging in health outreach. This is entirely optional, but helps us identify common experiences and pitfalls.**

* 20. **Finally, which of the following changes (if any) do you feel would benefit those communicating health the most? (check all that apply)**

- ☐ Increased support from professional bodies
- ☐ Increased support from academic institutions
- ☐ Increased support from advocacy / healthcare bodies
- ☐ Clearer guidelines and better training on public engagement
- ☐ Improved emotional support services
- ☐ Other (Please specify) _______________
- ☐ Practical legal advice
- ☐ More proactive stances from institutions and bodies
- ☐ Dedicated funding for outreach
- ☐ Better protective mechanisms from internet / social media companies
- ☐ None of the above

For any queries, please contact Dr. David Robert Grimes at d.r.grimes@qub.ac.uk or davidrobert.grimes@oncology.ox.ac.uk

For peer review only - http://bmjopen.bmj.com/site/about/guidelines.xhtml

Experiences in public communication of medical science & health

Thank you for taking part in this survey. Results will be treated anonymously, but if you are happy for us to contact you and perhaps quote from your experiences if appropriate, please indicate this below.

21. If you would like to be kept up to date on results and follow-up, please enter your e-mail address below.

22. We'd be grateful if you'd also supply your twitter handle. This will not be shared without your consent, and is entirely optional, but may prove useful in future work estimating network reach of health information.

For any queries, please contact Dr. David Robert Grimes at d.r.grimes@qub.ac.uk or davidrobert.grimes@oncology.ox.ac.uk

For peer review only - http://bmjopen.bmj.com/site/about/guidelines.xhtml

BMJ Open

Establishing a taxonomy of potential hazards associated with communicating medical science in the age of disinformation

Journal:	*BMJ Open*
Manuscript ID	bmjopen-2019-035626.R1
Article Type:	Original research
Date Submitted by the Author:	06-Feb-2020
Complete List of Authors:	Grimes, David; University of Oxford, & Queens University Belfast<br>Brennan, Laura ; Not applicable , Not applicable (patient advocate, deceased)<br>O'Connor, Robert; Irish Cancer Society
**Primary Subject Heading**:	Public health
Secondary Subject Heading:	Medical education and training
Keywords:	PUBLIC HEALTH, INFECTIOUS DISEASES, ONCOLOGY, MEDICAL JOURNALISM, MEDICAL EDUCATION & TRAINING

SCHOLARONE™
Manuscripts

I, the Submitting Author has the right to grant and does grant on behalf of all authors of the Work (as defined in the below author licence), an exclusive licence and/or a non-exclusive licence for contributions from authors who are: i) UK Crown employees; ii) where BMJ has agreed a CC-BY licence shall apply, and/or iii) in accordance with the terms applicable for US Federal Government officers or employees acting as part of their official duties; on a worldwide, perpetual, irrevocable, royalty-free basis to BMJ Publishing Group Ltd ("BMJ") its licensees and where the relevant Journal is co-owned by BMJ to the co-owners of the Journal, to publish the Work in this journal and any other BMJ products and to exploit all rights, as set out in our licence.

The Submitting Author accepts and understands that any supply made under these terms is made by BMJ to the Submitting Author unless you are acting as an employee on behalf of your employer or a postgraduate student of an affiliated institution which is paying any applicable article publishing charge ("APC") for Open Access articles. Where the Submitting Author wishes to make the Work available on an Open Access basis (and intends to pay the relevant APC), the terms of reuse of such Open Access shall be governed by a Creative Commons licence – details of these licences and which Creative Commons licence will apply to this Work are set out in our licence referred to above.

Other than as permitted in any relevant BMJ Author's Self Archiving Policies, I confirm this Work has not been accepted for publication elsewhere, is not being considered for publication elsewhere and does not duplicate material already published. I confirm all authors consent to publication of this Work and authorise the granting of this licence.

For peer review only

Establishing a taxonomy of potential hazards associated with communicating medical science in the age of disinformation

David Robert Grimes[1,2*] Laura J Brennan[†], and Robert O'Connor[3]

1. School of physical sciences, Dublin City University, Glasnevin, Dublin 9, Ireland
2. Department of Oncology, University of Oxford, Off Roosevelt Rd, Oxford, UK
3. Irish Cancer Society, Dublin 4, Dublin, Ireland

Correspondence: davidrobert.grimes@oncology.ox.ac.uk / davidrobert.grimes@dcu.ie

Abstract

Objectives: Disinformation on medical matters has become an increasing public health concern. Public engagement by scientists, clinicians, and patient advocates can contribute towards public understanding of medicine. However, depth of feeling on many issues (notably vaccination and cancer) can lead to adverse reactions for those communicating medical science, including vexatious interactions and targeted campaigns. Our objective in this work is to establish a taxonomy of common negative experiences encountered by those communicating medical science, and suggest guidelines so that they may be circumvented.

Design: We establish a taxonomy of the common negative experiences reported by those communicating medical science, informed by surveying medical science communicators with public platforms.

Participants: 142 prominent medical science communicators (defined as having >1000 Twitter followers and experience communicating medical science on social and traditional media platforms) were invited to take part in a survey, with 101 responses.

Results: 101 responses were analysed. Most participants experienced abusive behaviour (91.9%), including persistent harassment (69.3%) and physical violence and intimidation (5.9%). A substantial number (38.6%) received vexatious complaints to their employers, professional bodies, or legal intimidation. The majority (62.4%) reported negative mental health sequelae due to public outreach, including depression, anxiety, and stress. A significant minority (19.8%) were obligated to seek police advice or legal counsel due to actions associated with their outreach work. While the majority targeted with vexatious complaints felt supported by their employer / professional body, 32.4% reported neutral, poor or non-existent support.

Conclusions: Those engaging in public outreach of medical science are vulnerable to negative repercussions, and we suggest guidelines for professional bodies and organisations to remedy some of these impacts on front-line members.

Strengths and limitations

- Individuals involved in the communication of medical science were surveyed to ascertain their experiences in public outreach.
- Results of this survey established a taxonomy of adverse reactions those communicating medical science can encounter.
- Based upon survey feedback, suggested guidelines for professional bodies, research institutes, and medical centres to circumvent the worst impacts on members engaged in public outreach on medical science are preferred.
- Survey results should only be taken as indicative of the scope of the issue at this juncture.
- Much further research is needed to ascertain how the medical community can best act to counter the rise of medical disinformation whilst protecting practitioners

[†] Deceased 20th March 2019

For peer review only

Introduction

Despite being fundamental to societal well-being, many aspects of medical science remain poorly understood and frequently distrusted. Disinformation undermining health science and evidence-based medicine has increased markedly in the era of social media, and dangerous misconceptions abound, from perceived cancer risks and ostensible cures [1] to dangerous falsehoods about vaccination [2]. Improving public awareness and understanding of science and medicine is imperative if we are to maintain continued progress in research endeavours, and scientists, physicians, and science communicators have a crucial role to play in shaping public perceptions. Medical science is largely publicly funded, and direct communication of research with the wider public can be extraordinarily beneficial on a societal level. Accordingly, public engagement has become a prerequisite for many funding bodies. Informed engagement by patient advocates and media figures too can have marked impact on public understanding of medicine, empowering the public with facts with which to make important health decisions.

Improving public understanding of medical science is vital, as there many scenarios where public perception (or a vocal subset of that) is starkly at odds with scientific consensus. Frequently, medical science contradicts a narrative strongly held by particular groups within the wider public. For our purposes, we define a 'narrative' as a world-view or mindset shared by a given subgroup, which unifies that grouping. Narratives are often articles of faith, empowerment or comfort, frequently unsupported by available evidence or at odds with scientific consensus. For clarity, we concentrate herein on situations where there is no reputable evidence for a narrative, or where overwhelming scientific consensus is firmly against that viewpoint.

Misguided narratives can be supremely damaging, and the anti-vaccine movement is perhaps the most obvious example of this. Despite their life-saving efficacy of vaccination, opposition has existed since the time of Jenner [3]. The rise of social media has seen significant propagation of anti-vaccine narratives [4, 5, 6], driving uptake rates down and causing serious harm worldwide [7, 8]. In 2018, Europe saw the highest number of cases of measles cases in 20 years, numbering over 82,525 cases with at least 72 death - over fifteen-fold the figures from 2016 [9]. Such is the extent of the problem that in 2019 the World Health Organisation described vaccine hesitancy as a "Top ten threat to global health" [10]. Exposure to anti-vaccine conspiracy theory is a leading factor in parental intention to vaccinate [8], and evidence to date suggests that the deluge of vaccine disinformation across social media is extremely damaging to public understanding and health.

Other strongly-held narratives which clash with the weight of available scientific evidence include the claims propounded by the anti-fluoride movement [11, 12], the beliefs of the electromagnetic hypersensitivity movement [13], and the narratives of alternative medicine [14, 15]. Cancer patients are especially vulnerable to misinformation, and frequently targeted by charlatans and the misguided [16]. Consequences of this can be severe, with patients sometimes delaying or refusing conventional treatment. The net result of this is diminished survival statistics for those who engage with cancer pseudoscience, due to delayed treatment and sometimes rejection of conventional medicine. In some instances, subscribing to un-proven or disproven modalities could approximately half survival time [17].

While health falsehoods have always existed, the social media age has created new avenues for misinformation (misinformed advice) and disinformation (deliberate falsehoods) to propagate [16], rapidly bringing discredited ideas and dangerous pseudoscience to vast new audiences.

For peer review only

Scaremongering stories from dubious outlets propagate more readily than reliable fact-based information from reputable sources [18, 19]. In 2016, over half of all cancer stories shared on Facebook were medically unsound. Some have harnessed pseudoscience to sell questionable diets, supplements, and books, to the detriment of public understanding. Internet health guru Joseph Mercola, for example, made over $7 million in 2010 alone, proffering dubious treatments and advice [20], including denigration of conventional therapies for cancer. Mercola is far from unique, and such proclamations have huge potential for patient harm.

To overcome this challenge, public outreach by scientists, physicians, and evidence-based health advocates must be a crucial element to counter damaging fictions and empower our community with evidence-based information. A physician's recommendation, for example, is central to parental decisions to vaccinate [21]. Addressing patient concerns improves public health, and personal engagement by researchers and physicians can have a positive impact on public perception. Patient advocates and media figures have substantial ability to shift public perception; after Ireland saw HPV vaccine uptake drop from 87% to 51%, an alliance of healthcare professionals, researchers, and patient advocates were instrumental in countering the dominant falsehoods, and Ireland has seen a dramatic recovery in vaccine uptake rates [22]. To make inroads against the deluge of dubious health claims to which we're subjected, it's vital that scientists, clinicians, and patient groups must be on the vanguard of efforts to counter-act misinformation.

Those engaging in public outreach, however, often encounter enmity for publicly advocating scientific evidence. Scientific consensus often runs contrary to deeply-held beliefs, leading to certain groups attempting to undermine legitimate public scientific discourse. Motivations for this are multi-faceted, often depending on very specific circumstances. Conspiratorial thinking underpins many narratives, and those attempting to communication science are often vilified as "shills", or agents of a nebulous "Big Pharma". The phenomenon of identity protective cognition is also commonly encountered [23] and narrative believers frequently attacking those who cast doubt on their beliefs. Even when handled with sympathy and compassion, professional and patient advocates who challenge misconceptions can become targets for certain individuals and groups. These negative responses can range from verbal abuse to coordinated harassment campaigns, and even violence.

Aside from being deeply unsettling, such responses can cause professional and personal problems for those targeted. With the increasing emphasis on public engagement and ubiquity of the internet, this subject warrant urgent consideration, as there are currently few clear guidelines for researchers, clinicians, or patient advocates engaging in outreach work. Nor indeed is there a unified understanding of how adverse effects can manifest, and institutions and professional bodies are typically ill-equipped or muted in their support. This leaves those in the public eye or studying contentious topics vulnerable to vexatious complaints and even physical harm. Without awareness of this reality, a less than ideal situation where professional bodies can potentially be weaponised against researchers can too easily emerge. Accordingly, the aim of this work is to ascertain the typical experiences of those communicating medical science and identify how these might be counteracted.

Figure 1 A non-exhaustive taxonomy of negative experiences encountered by individuals engaging in public communication of health science. Subsections discussed in text.

For peer review only

Methods

Sample recruitment and selection criteria

The main recruitment fora for this study was online discussion groups for physicians, scientists, and patient advocates communicating aspects of medical science to the general public across social and traditional media. At the height of the Irish HPV vaccine confidence crisis [22], several physicians and scientists (based in Ireland and the UK) in these groups sought advice for negative experiences, including threats and malicious complaints to their employers and professional bodies, all of which were eventually dismissed. Group members across Europe echoed similar experiences in communicating vaccination science and in other health issues, and almost identical adverse reports came from colleagues across the America and Africa. Informal interviews were conducted on foot of this in these fora to identify common issues, as to the authors' knowledge there is no existent literature on the topic.

On basis of these interviews and related fora discussions, a survey was created, including free-form sections where subjects were free to expand on their own experiences. The wording of this survey is included in the supplementary material. The selection criteria was specifically for those communicating medical science both on social media (defined as having over 1000 followers on twitter) and in mainstream channels (defined as invited appearances on public television, radio, and / or in the form of newspaper articles & invited comment). From this, 142 individuals world-wide (from across Europe, America, Africa, and Asia) working predominantly in the English language were identified as fitting the criteria and invited to partake, of whom 101 (71.1%) responded. This survey was undertaken for indicative purposes and was collected from a non-randomised group with no expectation of transferable findings. Accordingly, it was thus exempt from requiring HRA / HRB approval, though informed consent was sought and obtained prior to subjects partaking, with all data appropriately anonymised. Subject details are given in table 1.

Patient and Public Involvement

As patient advocates play a substantial role to play in combatting misinformation on medical issues, several who met the inclusion criteria were invited to take part, with 15.8% of respondents being patient advocates. One co-author of this work (LBJ) was a prominent patient advocate.

Table 1: Participant details

Inclusion and completion	Invited to take part (N = 142)<br>Total Completed (N = 101)
**Gender**	Female (N=55)<br>Male (N=44)<br>Non-Binary / Undisclosed (N=2)
**Affiliation**	University / Medical Centre (N=52)<br>Unaffiliated (N=26)<br>Media organisation (N=20)<br>Charity (N=11)<br>Political organisation (N=4)
**Profession**	Medical Professional (N=23)<br>Scientist / Researcher (N=20)<br>Science communications (N=16)<br>Patient Advocate (N=16)<br>Health Policy (N=5)
**Years active**	Average: 10.7 years (Range 2-30 years)

Table 2 A non-exhaustive taxonomy of common adversarial tactics

**Misrepresentation of scientific evidence / expertise**		
Straw-manning	Misrepresenting scientific arguments to make them amenable to ridicule or attack, and to deflect or obscure evidence that undermines a particular narrative.	*"Mercury is toxic, yet scientists put it in vaccines!"* - This statement belies importance of dose and ignores the fact there is no evidence for harm from thimerosal in vaccines.
Cherry-picking / Quote-mining	Selective, manipulative filtering of scientific evidence or expert statements, taken out of context to undermine evidence-base or promote a narrative.	*"THC kills cancer, but doctors don't want you to know about cannabis cures."* - THC can kill cells in a petri dish, but killing plated cells is entirely different from treating human cancer.
Shifting the burden of proof	Insisting the onus is on scientists to 'disprove' claims rather than offering any evidence or rationale for assertions made.	*"GMOs are toxic, and scientists should prove us wrong"* - This assertion is untrue, and onus lies on those making the claim to proffer evidence for it.
**Discreditation attempts**		
Questioning qualification	Casting doubt on one's ability to question claims at hand. Typically, ostensible scepticism is not extended to claims supportive of the narrative.	*"This patient advocate isn't an expert, so they can't say this vaccine is safe!"* - One does not need to be an expert immunologist in this case to accurately reflect medical consensus.
Alleging vested interests	Claims that the speaker is compromised due to some apparent conflict of interest or that experts are otherwise lacking impartiality.	*"Who's paying you to say this?"* - Unsubstantiated allegation to deflect from absence of evidence for a narrative or claim.
Asserting Conspiracy theory	Allegations that the scientist, physician, or patient advocate is part of some conspiracy to suppress the truth or spread false information, either as a pawn or an active player.	*"She's part of a pharma cover-up to suppress natural cancer cures!"* - Appeals to conspiracy theory function to distract from lack of evidence.
**Dubious amplification of pseudoscientific narratives**		
Media Targeting	Targeting traditional or online media outlets to amplify dubious narratives, typically by-passing gatekeepers (science / health journalists etc) who would otherwise be more likely to spot pseudoscience.	Pitching dubious health claims to journalists as human interest stories - This approach was successfully used by anti-vaccine activists to push the dis credited link between autism and the MMR vaccine between 1998-2000.
Astroturfing / sock-puppeting	Use of fake social media accounts / fictitious pressure groups to provide an illusion of a wider grass-roots support for a particular narrative	Example: Accounts which spring up once an initial anti-fact site is blocked but which include misinformation consistent with the originator's initial social media accounts.
Celebrity endorsement	Celebrities and influencers can have disproportionately large impact on the perception of public even in areas where they have no relevant expertise or knowledge.	There are numerous examples of this, especially in relation to anti-vaccine activism, including actors and models being cited for their purported knowledge of complex health issues.
**Malicious complaints / abuse of regulatory frameworks**		
Poisoning the well / Smear campaigns	The spreading of malicious claims regarding an individual's professional or personal conduct to undermine them or discourage others from engaging with them.	*"I've heard that doctor abuses patients"* - Inflammatory slurs such as these are designed to discredit, and are not in any way substantiated, but calculated to invoke disgust or contempt.
Vexatious complaints to employers	Making calculated complaints to one's employer or threatening to do so in order to intimidate them into silence.	Exaggerated / misleading accounts of interactions with public advocates and demands to censure them, typically aimed at an individual's university or employer.
Vexatious complaints to regulatory bodies	Abusing procedures of professional bodies to target a researcher / medic who presents a challenge to a narrative. These may also include unwarranted freedom of information requests or vexatious parliamentary questions.	Registering complaints with a medical regulatory body against a doctor for their advocacy of evidence-based positions. Physicians especially vulnerable, as typically all complaints must be investigated, regardless of merit.
**Intimidation**		
Harassment / abuse	Harassment can take many forms, and personal abuse is perhaps most common. Threats (implied and direct) are often employed.	Abusive language made publicly or in direct messages, and posting of private contact details, phone numbers, addresses etc.
Legal threats	Legal notices and mechanisms can also be utilised to silence researchers questioning a narrative, from cease and desist notices to defamation claims.	Threatening to bring an advocate to court for alleged defamation, also used judiciously to limit independent investigation on pseudoscientific narratives
Physical intimidation	Implicit or explicit threats of physical violence is an u~~U~~nfortunately, potent method of intimidating scientists into silence. This includes threats of physical violence or rape (the latter usually directed at female discussants)	Stalking of private abodes, explicit threats, or actions like spitting etc. There are instances of security being required for scientific meetings on publicly contentious subjects, due to implications of or threats of violence.

Results

142 individuals were approached to undertake the survey, with 101 responding (response rate: 71.1%). In addition to survey questions on known problems, participants were also invited to expand on noteworthy negative situations they may have encountered while engaging in health outreach. This section was entirely optional, and 53 participants (52.5% of subjects) opted to share their experiences. Example responses are shown below; please note that some responses have been edited or partially redacted to exclude potentially identifying information and preserve anonymity.

"Accusations - including by one Senator - that [we are] uncaring, dismissive, neglectful, arrogant, or paid by pharma companies when advocating for vaccines." (Misrepresentation)

"I find my expertise is questioned - this often seems to be when men find it difficult to accept women with intelligence and qualifications. Sexist insults are a typical go-to response." (Discreditation)

"The worst one that hurt me professionally and personally was that activists gathered my emails using [Freedom of Information Requests] and handed chosen packets of them with a story to different reporters." (Misrepresentation / Discreditation / Dubious Amplification)

"Persistent negative comments on twitter; usually it doesn't last long but it can feel very intense while it's happening!" (Intimidation)

"I have been served with a SLAPP lawsuit in order to silence my outreach work. Frequently receive harassing emails, malicious comments made on blog." (Malicious Complaints)

"Social media co-ordinated intimidation, implied threats of legal action (for defamation). Mocking, undermining, condescension and attacks for being an industry shill, although.. I am just a patient advocate. Being called a liar, that I never had cancer, that I deserved cancer due to my attitude, that I have been mutilated by conventional medical treatment, and that I am no longer a woman (having had mastectomy for cancer). That my cancer will return and I deserve that." (Dubious Amplification / Misrepresentation / Discreditation)

"I have had anti-vaccine organizations and individuals attempt to prevent my public appearances and have been the subject of numerous online smear campaigns accusing me of being 'a shill for Big Pharma' etc." (Discreditation / Dubious Amplification)

"Those who attack me very frequently try to do it by targeting me at my job, sending bogus complaints to my bosses and the university. From my observation, that is the go-to attack, the first thing these groups do." (Malicious Complaints)

"I had to contact the police, who visited the person who was harassing me. I also involved social services. We bought a CCTV to monitor our front door after a strange envelope was hand delivered. The person involved has targeted several people before and continues to target individuals who advocate vaccination." (Intimidation)

"Abuse and accusations of corruption are the most common adverse reaction I get. Sometimes a particular group petition one's employer and try to create trouble for them. I have been lucky in the past when this happened to have had supportive universities who appreciate my outreach work. I have

For peer review only

in the past had slightly unhinged individuals writing rambling, implicitly threatening letters to my office which ultimately required police intervention." (Discreditation / Malicious Complaints / Intimidation)

"The worst are gendered insults (being called a cunt, etc.) and rape/death threats. I have had one empty legal threat that was widely publicized." (Intimidation / Malicious Complaints)

"Regular threats to sue for defamation." (Malicious Complaints)

"Attempts to get me fired, public records act requests for emails, verbal attacks on my children." (Malicious Complaints, Intimidation)

"One of the most unpleasant things is that certain people or groupings will use very underhanded tactics to respond to perceived criticism. If they can't refute the science, it isn't uncommon for them to go after you personally, alleging all manner of things to anyone who'll listen; that you're incompetent, or unethical, or perverted. It seems they throw things wildly to see what sticks, but it can be extraordinarily unpleasant to endure." (Dubious Amplification, Discreditation)

"My main concern has been obsessed individuals who declare their enmity and seem to be unconstrained by civil norms." (Intimidation)

"Homeopathy advocates looked up my Linkedin profile and called my employer to complain about my comments on the radio. My employer did not support me and I ended up having to stop the activity I had been planning." (Misrepresentation / Malicious Complaints)

"Being threatened with physical violence" (Intimidation)

"A delusional supporter of [an individual] I wrote about accused me and my lawyer of stalking him and killing his in-laws. He sent accusing emails to the faculty of my school and all the police departments in my state. [They] also accused me of being a terrorist and complained about me to the FBIs Terrorism Joint Task Force. That gave me many nervous, sleepless nights." (Discreditation / Malicious Complaints)

"Death threats received, employer unhelpful, sorted myself" (Intimidation)

"I haven't experienced many negative encounters because I would say I am only lightly involved in public engagement. However the reason I don't become more heavily involved in this area is fear of this kind of abuse and vexatious complaints to my employer or regulatory body." (Malicious Complaints)

Based upon this and survey responses, a non-exhaustive taxonomy was constructed detailing common experiences of those communicating medical science to the public. While non-exhaustive, it forms a useful basis for more systematic investigation. Adversarial experiences in communicating medicine were broadly stratified into five distinct classes, illustrated graphically in figure 1, with these sub-types detailed in detail in table 2.

Participant details are given in table 2. Topics covered / channels of engagement and fora for abusive interactions are depicted in figure 2, informed by survey questions 4,5, and 16. Twitter is disproportionately represented, as prominence on that platform was part of the selection criteria. Other fora cited included books, documentaries, newspapers, podcasts - and in one instance criticism under parliamentary privilege. The vast majority of those surveyed (N = 94, 93.1%) reported being the recipient of personal abuse of professional smears in the course of their outreach efforts. A majority

For peer review only

(N = 70, 69.3%, survey question 13) had experienced targeted abuse from at least one particular grouping. The most common groupings for negative reactions were anti-vaccine and alternative medicine groups respectively (N = 43 each, 42.6%) followed by dietary advocates (N = 26, 25.7%), 'wellness' groups (N = 17, 16.8%), religious and chronic illness groups (both N = 15, 14.9%), anti-fluoride and autism focused groups (both N =12, 11.9%). Others cited by 3 or less respondents included electromagnetic hypersensitivity factions, conspiracy theorists and anti-GMO organisations.

Figure 3 depicts types of experiences reported by participants, ranging from the relatively minor to the severe, informed by survey responses to questions 11, 12, and 14. Of participants surveyed, a majority (N = 63, 62.4%) reported fallout from public engagement had caused them at least some negative mental health sequelae, including depression, anxiety, and stress. Most of this was reported as minor, but considerable or significant mental health ramifications were reported by 15 respondents (14.9%). Of the participants, 20 (19.8%) were obligated to seek police advice / legal counsel as a direct result of targeted actions associated with their outreach work. Of those receiving vexatious complaints (N = 39, 38.6% of all respondents), most (67.6%) felt supported or well supported by their institution, employer, or professional body, with whilst 16.2% deemed support to be neutral, with an equal number (16.2%) feeling poorly or entirely unsupported. Predictably perhaps, gender specific abuse was far more likely to be directed at women (40% of female respondents) than men (6.8% of male respondents), with this difference being highly significant ($p << 0.001$, calculated by Welch's t-test)

Table 3 depicts frequency of different experiences (positive and negative) reported by respondents, taken from data in survey question 8. In response to survey question 9, 29.7% (N=30) responded that they found outreach largely rewarding, 38.6% rewarding (N=39), 29.7% mixed (N=30), and ~2% not very rewarding (N=2). Changes respondents felt would most improve outreach work is depicted in figure 4 (from survey question 20). Free-form responses to under category of "other" included Improving media science literacy (false balance and issues with platforming of anti-science views were repeatedly mentioned), legal defence funds, better coordination of professional bodies, robust infrastructure on social media to report disinformation, and better support from police organisations.

Table 3: Frequency of experiences with outreach

Statement	Always	Frequently	Sometimes	Infrequently	Never	Unsure
Engagement is mutually informative	6	31	52	10	2	0
Engagement changes minds & informs	1	22	64	7	1	6
My contributions are welcome & appreciated	1	56	39	3	0	0
My efforts contribute to public understanding	4	47	49	1	0	0
My efforts are taken in good faith	3	51	38	6	0	3
My efforts feel futile	1	9	50	34	7	0
Engagement takes a toll on mental health	2	12	44	26	16	0

Figure 2 (a) Topics covered by participants (b) channels of engagement for subjects surveyed (c) Fora for negative interactions

Figure 3 Proportion of negative experiences recorded including (a) abuse experienced (b) violence and intimidation (c) vexatious complaints.

Figure 4 Changes respondents deemed most likely to benefit medical science communication the most.

For peer review only

Discussion

In a globalised information age, medical science can appear disconnected and aloof from those it serves to help. Educational and professional bodies (including universities and medical centres) have a unique societal role to inform their peers and public on evidence-based medicine, and a responsibility to adjust to modern communications realities. We can collectively no longer remain on the fence in supporting health information advocacy. While being mindful not to over-infer from our survey, we can use it as a basis to make some suggestions. It is vital to have proactive strategies in place to support those engaging in medical outreach, and to maintain a high calibre for public discussion. It is also crucial that those engaging in outreach are cognizant of the potential pitfalls, and afforded ample support. Given the gendered nature of much of the abuse reported, it seems likely that the hostile environment encountered online could dissuade many talented female communicators from engaging fully, to focus on but one example. It is also important to note that despite the sometimes fraught nature of medical science outreach, a majority of respondents (68.2%, N = 69) found the undertakings rewarding or very rewarding. More can be done to avoid common pitfalls of this endeavour, however, much of which is outlined in this manuscript.

One potential weakness of the survey is the potential for ambiguous definition. Alternative medicine, for example, is typically defined as ostensible medical interventions for which there is insufficient or disconfirmatory evidence; for example, the National Science Board define it as referring to "all treatments that have not been proven effective using scientific methods". As no specific definition was given in the survey text, it is possible respondents substituted their own meaning to some extent. As those surveyed were drawn from science communicators with significant media profiles however, it might be expected that their definitions were more unified that a typical respondent might be. There is also some unavoidable ambiguity with terms such as "abuse" and "smears". There is a level of subjectivity to these terms, which respondents were left to define themselves. This renders the responses potentially subjective, although the free-form responses do indicate behaviours that could be seen as objectively abusive.

There is also a serious point that must be at least considered – that advocates for medical science may on occasion engage in ill-advised tactics or unhelpful rhetoric. Nor does one's expertise render them infallible, and it is certainly possible that advocates for science might sometimes engage in a counterproductive fashion. To ascertain this requires some context and nuance, especially for academic and medical institutions whose staff might be the subject of complaints. But rather than be reactionary, it is imperative that bodies and institutions have robust policy for dealing with issues that might arise, both so they can correct errant behaviour by members and so that they cannot be weaponised by malicious complaints. Based on the feedback to this survey and wider discussion on the topic, we offer the following suggestions to employers and professional bodies whose members might engage in the communication of medical science. While by no means comprehensive, the following guidelines might be beneficial towards establishing policy for dealing with issues that can arise.

Suggested guidelines for professional bodies and employers

1. Educational / professional organisations must recognise a commitment to support evidence-based actions by their members. This may require oversight of such activities and investment in the governance / training resources to protect members willing to act as advocates.

For peer review only - http://bmjopen.bmj.com/site/about/guidelines.xhtml

2. Institutions and professional bodies should have robust measures in place to oversee communication activities associated with their members, and to make assessments as to whether individuals are communicating established facts in good faith or are contributing to undermining of fact with potential legal / reputational damage.

3. When institutions receive complaints regarding members, the subject must be afforded presumption of innocence rather than being served with reactionary and inflexible procedure, lest the institution might become an unwilling tool against science.

4. In case of disputes and complaints, competent and impartial individuals should be engaged to independently assess complaints, cognisant of vital background and context.

5. Coordination between press offices and those engaged in outreach would improve communication, pre-emptively identifying those likely to be targets for malicious tactics.

6. Support for those identified as being on engagement frontlines should be maintained, with clear legal advice / institutional support for targeted members.

7. Organisations must be vocal in supporting public-facing members, willing to issue strong rebuttals of vexatious complaints against individuals.

8. Professional bodies and employers should promote strive to promote both scientific freedom of speech and strive to champion evidence-based advocacy.

9. When possible, those expected to engage in outreach should be trained in methods that reduce opportunities for personal and professional attacks.

Conclusions

The question of how we best communicate health science in the modern era is an area where more research is urgently required, especially on the role of social media, and optimum ways physicians, researchers, and other public-facing figures can promote good medical science and mitigate falsehoods. The suggestions herein ought to be taken as a starting point, with discussion evolving as improved evidence materialises. There are wider problems implicit in all this those communicating science cannot tackle in isolation; social media regulation particularly is a serious issue, both in regards to the spreading of misinformation / dis-information, and with respect to procedures preventing the potential weaponisation of social media platforms. Social media platforms must ultimately be made answerable to regulatory over-sight, just as every other important aspect of life is; claims of innocence are unconvincing when their business model is so clearly dependent on advertising engagement at the cost of lives. The problem of poor reporting and false balance [24] in conventional media outlets also must be considered, and there is significant scope for scientists and doctors to contribute to policy in these areas. There is ample evidence that physicians and scientists have an important role to play in combatting health disinformation, as has recently been argued by one of the authors in relation to vaccination for British Medical Journal opinion [25]. But equally, it is crucial that those engaging in this vital work have the requisite support from their institutions, so that deleterious consequences of laudable outreach work might be circumvented. It is increasingly clear that disinformation about medicine and illness has become ubiquitous, with severe consequence for both our collective health and public understanding of medical science. Scientists and physicians must be at the vanguard of the pushback against these dangerous falsehoods – our societal well-being depends upon it.

For peer review only

Electronic Supplementary Material

A full version of the questionnaire and further analysis can be found in the electronic supplementary material.

Acknowledgements

The authors would like to thank the scientists, physicians, and patient advocates who gave their time to discuss the issues they face in communicating science, and for sharing their insights, and the reviewers for their useful recommendations. In particular, the authors would like to profoundly thank Dr Laura J Brennan for her invaluable input; Laura was present and helped conceive the concept from which this paper sprung. She was a passionate advocate of evidence-based science communication and especially for the HPV vaccine, championing it despite enduring a life-limiting and difficult cervical cancer diagnosis. Following a confidence crisis in the vaccine in Ireland driven by anti-vaccine activism, her tireless work was a substantial factor in reversing the damage wrought by disinformation. Laura passed away 20th March 2019, but while we have lost a friend and colleague, hers is a legacy that will resonate for generations. Laura embodied precisely how vital health communication is to promoting public health; Ireland, and the world, owe her a huge debt, and we dedicate this work to her memory.

Contributor statement

DRG, LJB & ROC conceived the concept for this paper, and worked on survey design. DRG & ROC performed the analysis and wrote the manuscript.

Competing interests

The authors declare that they have no competing interests.

Funding statement

The authors declare that they have no specific funding for this work.

Data Sharing

Full survey forms available in electronic supplementary material.

References

1. Shahab, L., McGowan, J.A., Waller, J., Smith, S.G.: Prevalence of beliefs about actual and mythical causes of cancer and their association with socio-demographic and health-related characteristics: Findings from a cross-sectional survey in england. European Journal of Cancer 103, 308{316 (2018)

2. Kata, A.: A postmodern pandora's box: anti-vaccination misinformation on the internet. Vaccine 28(7), 1709-1716 (2010)

For peer review only

3. Poland, G.A., Jacobson, R.M.: The age-old struggle against the antivaccinationists. New England Journal of Medicine 364(2), 97{99 (2011)

4. Zimmerman, R.K., Wolfe, R.M., Fox, D.E., Fox, J.R., Nowalk, M.P., Troy, J.A., Sharp, L.K.: Vaccine criticism on the world wide web. Journal of medical internet research 7(2), 17 (2005)

5. Kata, A.: Anti-vaccine activists, web 2.0, and the postmodern paradigm{an overview of tactics and tropes used online by the anti-vaccination movement. Vaccine 30(25), 3778{3789 (2012)

6. Grimes, D.R.: On the viability of conspiratorial beliefs. PloS one 11(1), 0147905 (2016)

7. Goertzel, T.: Conspiracy theories in science. EMBO reports 11(7), 493{499 (2010)

8. Jolley, D., Douglas, K.M.: The e ects of anti-vaccine conspiracy theories on vaccination intentions. PloS one 9(2), 89177 (2014)

9. Thornton, J.: Measles cases in Europe tripled from 2017 to 2018. British Medical Journal Publishing Group (2019)

10. Organization, W.H., et al.: Ten threats to global health in 2019. 2019. Retrieved January (2019)

11. Freeze, R.A., Lehr, J.H.: The Fluoride Wars: How a Modest Public Health Measure Became America's Longest-Running Political Melodrama. John Wiley & Sons, ??? (2009)

12. Grimes, D.R.: Commentary on "are fluoride levels in drinking water associated with hypothyroidism prevalence in england? a large observational study of gp practice data and fluoride levels in drinking water". J Epidemiol Community Health 69(7), 616{616 (2015)

13. Grimes, D.R., Bishop, D.V.: Distinguishing polemic from commentary in science: Some guidelines illustrated with the case of sage and burgio (2017). Child development 89(1), 141{147 (2018)

14. Singh, S., Ernst, E.: Trick or Treatment: The Undeniable Facts About Alternative Medicine. WW Norton & Company, (2008)

15. Grimes, D.R.: Proposed mechanisms for homeopathy are physically impossible. Focus on Alternative and Complementary Therapies 17(3), 149{155 (2012)

16. The, L.O.: Oncology," fake" news, and legal liability. The Lancet. Oncology 19(9), 1135 (2018)

17. Johnson, S.B., Park, H.S., Gross, C.P., James, B.Y.: Complementary medicine, refusal of conventional cancer therapy, and survival among patients with curable cancers. JAMA oncology 4(10), 1375{1381 (2018)

18. Vosoughi, S., Roy, D., Aral, S.: The spread of true and false news online. Science 359(6380), 1146{1151 (2018)

19. Brady, W.J., Wills, J.A., Jost, J.T., Tucker, J.A., Van Bavel, J.J.: Emotion shapes the diffusion of moralized content in social networks. Proceedings of the National Academy of Sciences 114(28), 7313{7318 (2017)

20. Smith, B.: Dr. mercola: Visionary or quack? Chicago (2012)

21. Rosenthal, S., Weiss, T.W., Zimet, G.D., Ma, L., Good, M., Vichnin, M.: Predictors of hpv vaccine uptake among women aged 19-26: importance of a physician's recommendation. Vaccine 29(5), 890{895 (2011)

22. Corcoran, B., Clarke, A., Barrett, T.: Rapid response to hpv vaccination crisis in ireland. The Lancet 391(10135), 2103 (2018)

23. Kahan, D.M.: Misconceptions, misinformation, and the logic of identity-protective cognition (2017)

24. Grimes, D.R.: A dangerous balancing act. EMBO reports (2019)

25. Grimes, D.R.: "David Robert Grimes: Vaccines-How Can We Counter Misinformation Online?" The BMJ, 7 Nov. 2019, blogs.bmj.com/bmj/2019/11/06/david-robert-grimes-vaccines-how-can-we-counter-misinformation-online/.

For peer review only

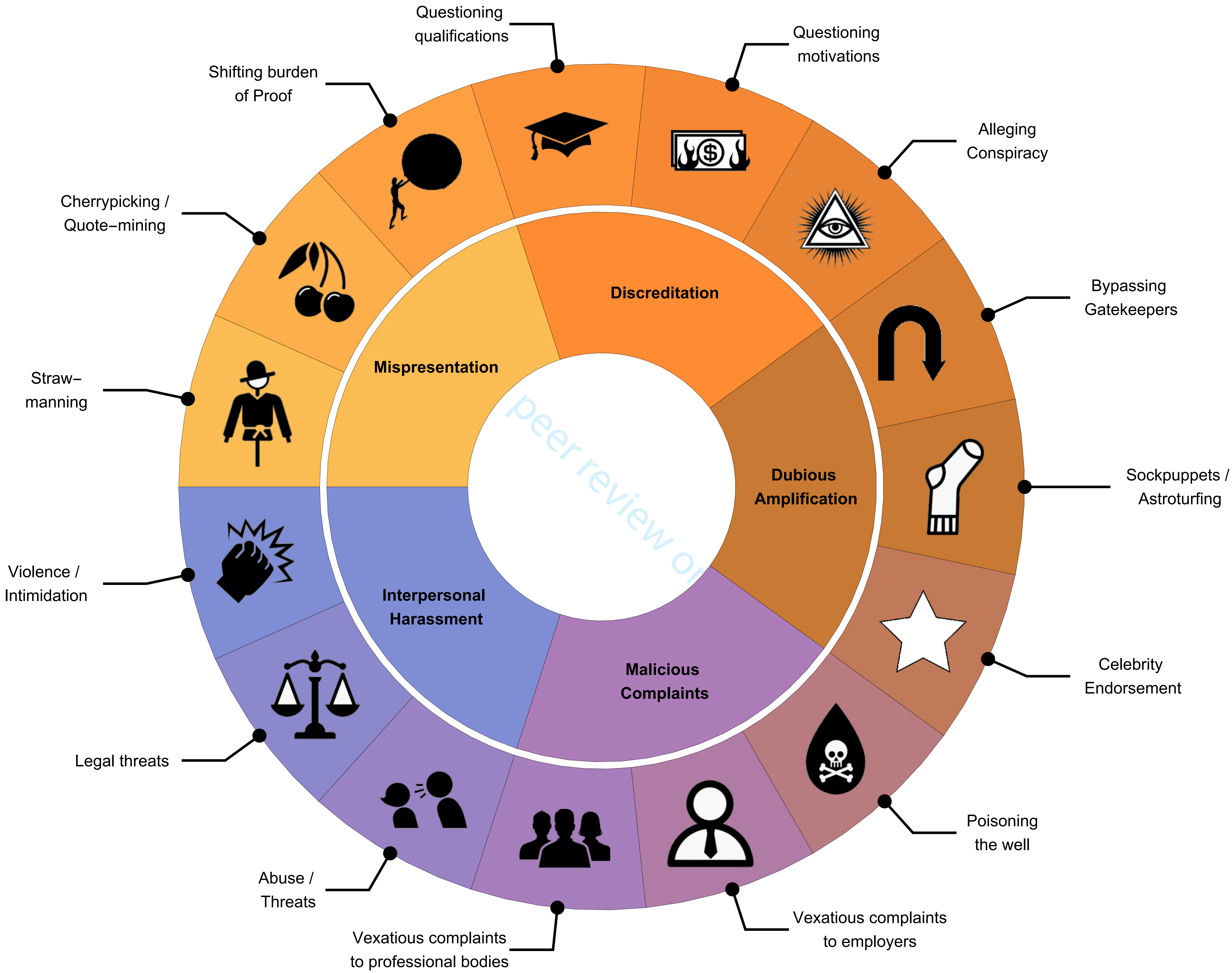
Questioning qualifications
Questioning motivations
Shifting burden of Proof
Alleging Conspiracy
Cherrypicking / Quote–mining
Bypassing Gatekeepers
Discreditation
Mispresentation
Straw–manning
Dubious Amplification
Sockpuppets / Astroturfing
Violence / Intimidation
Interpersonal Harassment
Malicious Complaints
Celebrity Endorsement
Legal threats
Poisoning the well
Abuse / Threats
Vexatious complaints to employers
Vexatious complaints to professional bodies

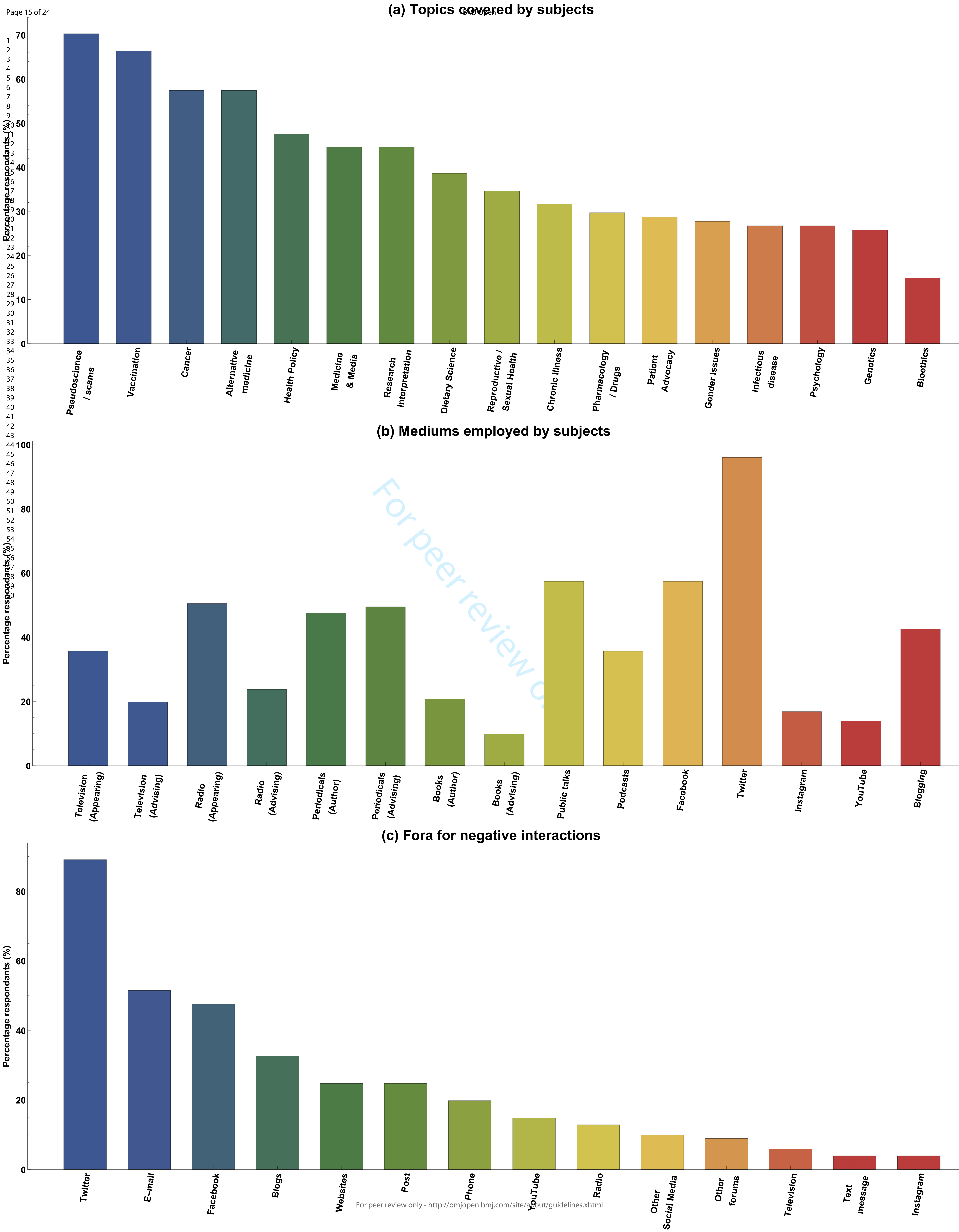

For peer review only

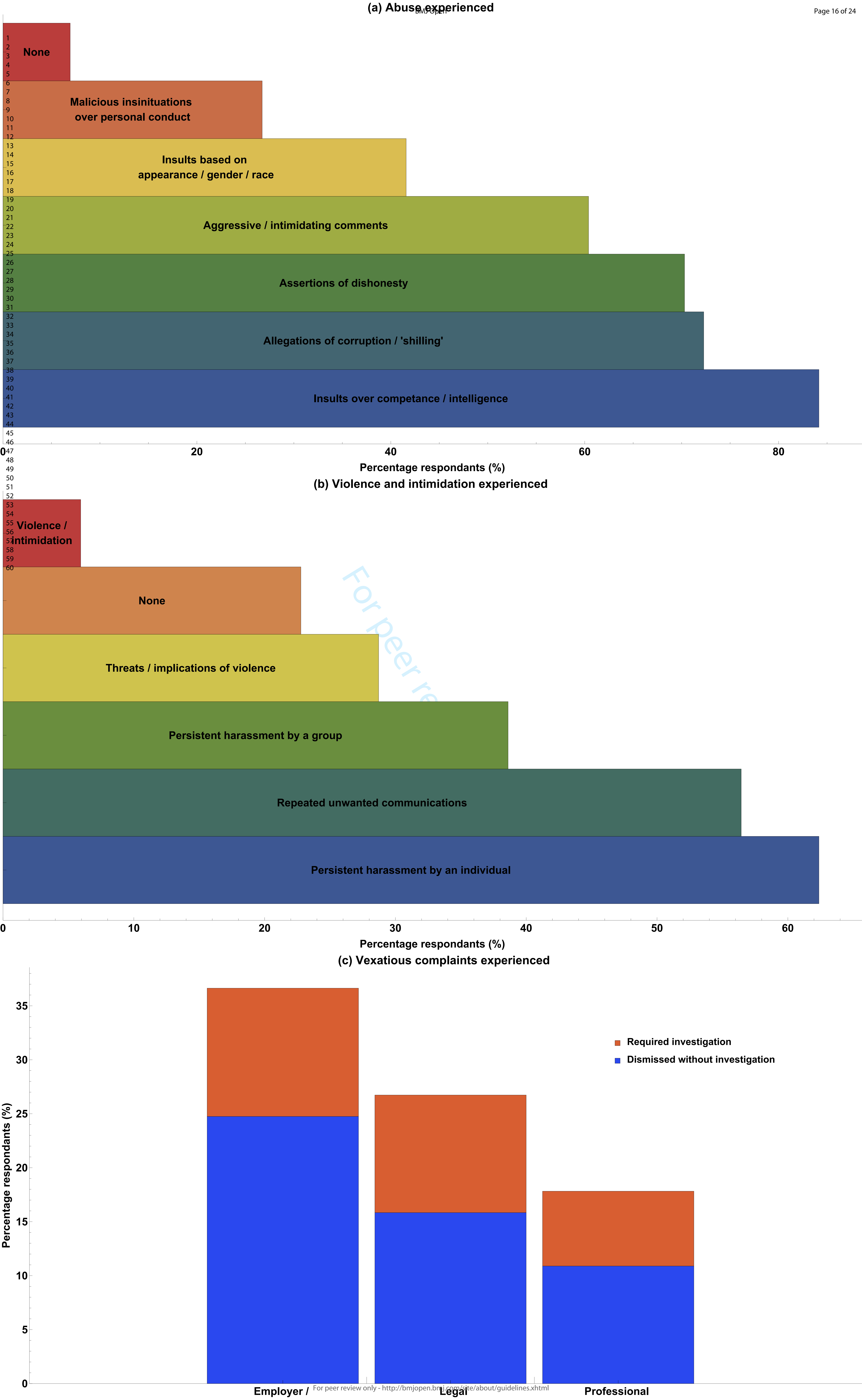

For peer review only - http://bmjopen.bmj.com/site/about/guidelines.xhtml

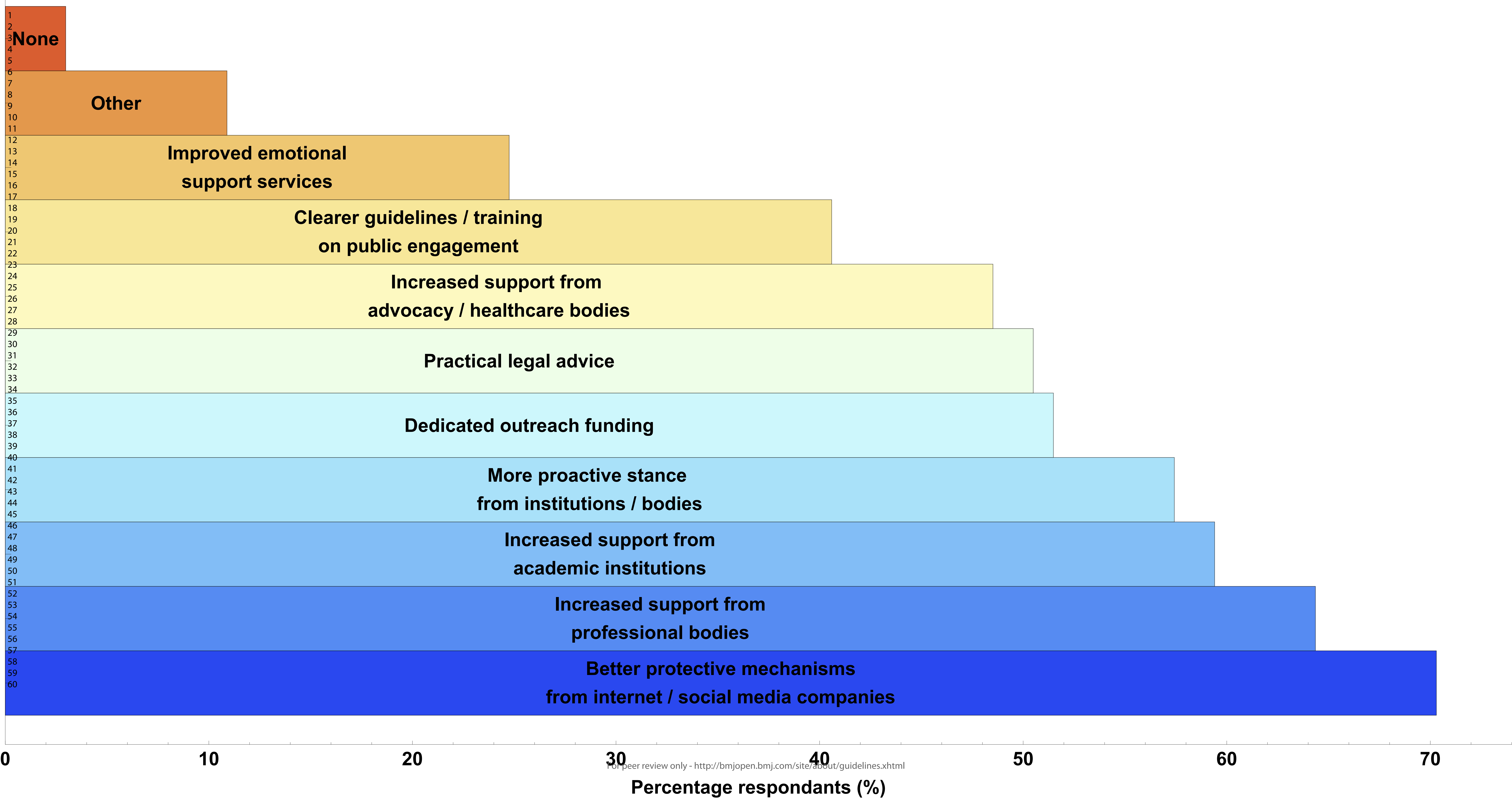

For peer review only - http://bmjopen.bmj.com/site/about/guidelines.xhtml

* Required Information

Experiences in public communication of medical science & health

This survey is part of a research project that aims to better understand the experiences of medical professionals, scientific researchers, patients advocates, health journalists, & policy makers in communicating health issues to the public. It consists of 20 short questions, & should take 5-10 minutes to complete. All responses are anonymous, and their content will be used for research purposes only. If you might be willing to contribute more about your experiences we invite you to inform us of this on the final page of the survey. For any questions, please e-mail d.r.grimes@qub.ac.uk

*** 1. Please select that which best describes your primary role (Select one option)**

- ○ Medical Professional
- ○ Scientific Communications
- ○ Scientist / Researcher
- ○ Health Journalism
- ○ Patient Advocate
- ○ Health Policy
- ○ Other (Please specify) __________

*** 2. Please specify your gender (Select one option)**

- ○ Female
- ○ Non-Binary
- ○ Male
- ○ Prefer not to say

*** 3. Please indicate which of the following organsiations you are professionally affiliated with that are directly relevant to your outreach efforts.**

- ☐ University / research institute
- ☐ Media outlet
- ☐ Medical institute
- ☐ Political grouping
- ☐ NGO / Charity
- ☐ Non-affiliated (Patient Advocate / independent)
- ☐ Other (Please specify) ______________

*** 4. Please select the topic or topics you frequently communicate about with the public. Please note that this includes topics you might debunk or criticize. Select all that apply.**

- ☐ Vaccination
- ☐ Alternative Medicine
- ☐ Medicine in media
- ☐ Dietary Science
- ☐ Pseudoscience / scams
- ☐ Medical science
- ☐ Cancer
- ☐ Chronic illness
- ☐ Bioethics
- ☐ Health Policy
- ☐ Infectious diseases
- ☐ Interpretation of research
- ☐ Reproductive / Sexual Health
- ☐ Pharmacology / drugs
- ☐ Gender issues
- ☐ Patient Advocacy
- ☐ Psychological issues
- ☐ Genetics
- ☐ Other (Please specify) ______________

*** 5. What media do you use for public engagement? Select all that apply.**

- ☐ Television (appearances)
- ☐ Television (advising / quoted)
- ☐ Radio (appearances)
- ☐ Radio (advising / quoted)
- ☐ Newspapers / Magazines (Author)
- ☐ Newspapers / Magazines (advising / quoted)
- ☐ Books (Author)
- ☐ Books (Advising / quoted)
- ☐ Public talks
- ☐ Podcasts
- ☐ Facebook
- ☐ Twitter
- ☐ Instagram
- ☐ Youtube
- ☐ Blogging
- ☐ Other (Please specify) _______________

*** 6. Approximately how long have you been communicating medical issues to the public? Please round to the nearest whole year**
(Enter a value between 0 and 120)

____

*** 7. Please select what best describes the extent of your public engagement (Select one option)**

- ○ I am heavily involved in public outreach
- ○ I am considerably involved in public outreach
- ○ I am involved in public outreach
- ○ I am lightly involved in public outreach
- ○ My vocation is media or outreach centred
- ○ Other (Please specify) ___________

For any queries, please contact Dr. David Robert Grimes at d.r.grimes@qub.ac.uk or davidrobert.grimes@oncology.ox.ac.uk
For peer review only - http://bmjopen.bmj.com/site/about/guidelines.xhtml

Experiences in public communication of medical science & health

Part II: Engagement experiences

8. In your opinion, how frequently do the following sentiments apply to your experiences of public engagement?

	Always	Frequently	Sometimes	Infrequently	Never	Unsure
*(a) I feel my efforts contribute to public understanding	○	○	○	○	○	○
*(b) I find engagement prompts constructive and respectful discussion	○	○	○	○	○	○
*(c) Engagement is mutually informative	○	○	○	○	○	○
*(d) My contributions are welcomed & appreciated	○	○	○	○	○	○
*(e) Engagement increases my personal understanding	○	○	○	○	○	○
*(f) I feel I correct some misconceptions	○	○	○	○	○	○
*(g) I find my engagement changes minds & informs	○	○	○	○	○	○
*(h) I consider outreach personally rewarding	○	○	○	○	○	○
*(i) My efforts feel futile	○	○	○	○	○	○
*(j) Engagement takes a toll on my mental health & well-being	○	○	○	○	○	○
*(k) I find my efforts are taken in good faith by others	○	○	○	○	○	○

*** 9. Please indicate whether you have experienced any of the following negative experiences during your engagement efforts. Select all that apply. Note that for the purposes of this survey, vexatious complaints are defined as those raised chiefly to harass or intimidate.**

- ☐ Personal abuse
- ☐ Personal smears
- ☐ Professional smears
- ☐ Physical threats
- ☐ Threats of sexual violence
- ☐ Harassment from individuals
- ☐ Coordinated harassment from specific groups
- ☐ Questioning of one's motivations
- ☐ Other (Please specify) _______________
- ☐ Sexist or gender-specific abuse
- ☐ Racist of ethnic-specific abuse
- ☐ Intimidation attempts
- ☐ Vexatious legal threats
- ☐ Vexatious complaints to employers
- ☐ Vexatious complaints to professional bodies
- ☐ Persistent unwanted communication
- ☐ No negative experiences

*** 10. How would you best describe your feelings about your outreach experiences to date? (Select one option)**

- ○ Largely rewarding
- ○ Rewarding
- ○ Mixed
- ○ Not very rewarding
- ○ Not at all rewarding
- ○ Other (Please specify) ___________

For any queries, please contact Dr. David Robert Grimes at d.r.grimes@qub.ac.uk or davidrobert.grimes@oncology.ox.ac.uk

For peer review only - http://bmjopen.bmj.com/site/about/guidelines.xhtml

Experiences in public communication of medical science & health

*** 11. If you have been the recipient of abuse or personal / professional smears stemming from your engagement, please indicate which of the following you've experienced (check all that apply)**

- ☐ Aggressive or intimidating comments
- ☐ Insults based on appearance, race, or gender
- ☐ Malicious comments about one's motivations / allegations of 'corruption' or 'shilling'
- ☐ Malacious comments about one's personal or sexual conduct
- ☐ Other (Please specify) ______________
- ☐ Assertions that you are dishonest or deliberately misleading
- ☐ Insults about your professional competence or intelligence
- ☐ I have not experienced any of the above

*** 12. Please indicate whether you have been subjected to any of the following (check all that apply)**

- ☐ Threats or implications of physical violence
- ☐ Physical violence or intimidation
- ☐ Spreading of malicious rumours
- ☐ Repeated unwanted communications
- ☐ Other (Please specify) ______________
- ☐ Persistent trolling / harassment by an individual
- ☐ Persistent trolling / harassment by a group
- ☐ None of the above

*** 13. If you have been the victim of targeted abuse for your outreach work by a group or community, what best describes that grouping? Choose all that apply if relevant**

- ☐ Anti-vaccine groups
- ☐ Dietary advocates
- ☐ Chronic illness groups
- ☐ Anti-fluoride groups
- ☐ Electromagnetic hypersensitivity groups
- ☐ Other (Please elaborate as appropriate) ______________
- ☐ Religious groupings
- ☐ Alternative medicine advocates
- ☐ "Wellness" groups
- ☐ Autism-focused groups
- ☐ I have not encountered this

*** 14. Please indicate whether you have experienced any of the following. Note that for the purposes of this survey, vexatious complaints are defined as those raised chiefly to harass or intimidate.**

- ☐ Vexatious complaint to employer or institution - complaint dismissed without investigation
- ☐ Vexatious complaint to employer or institution - required investigation
- ☐ Vexatious complaint to a professional body - complaint dismissed without investigation
- ☐ Vexatious complaint to a professional body - required investigation
- ☐ Vexatious legal complaint - complaint dismissed without investigation
- ☐ Vexatious legal complaint - complaint required investigation
- ☐ None of the above

*** 15. If you have experienced a vexatious complaint, did you feel supported by your institution or professional body? (Select one option)**

- ◯ Highly supported
- ◯ Supported
- ◯ Neutral
- ◯ Not well supported
- ◯ Entirely unsupported
- ◯ Not applicable

*** 16. If you have experienced abusive or diminishing comments, in which fora are these typically delivered or shared? (check all that apply)**

- ☐ E-mail
- ☐ Post
- ☐ Phone calls
- ☐ Texts
- ☐ Twitter
- ☐ Facebook
- ☐ Instagram
- ☐ Other (Please specify) _______________
- ☐ Youtube
- ☐ Other social media
- ☐ Blogs
- ☐ Websites
- ☐ Public television
- ☐ Public radio
- ☐ Not applicable

*** 17. Have you ever had to take legal action or consult law-enforcement officials regarding malicious communications, threats, or claims? (Select one option)**

- ◯ Yes
- ◯ No
- ◯ Not applicable
- ◯ Prefer not to say

*** 18. Have negative reactions to public engagement ever caused you mental health problems (depression, anxiety, stress, etc) or otherwise impeded your functioning? (Select one option)**

- ◯ Yes - severely
- ◯ Yes - considerably
- ◯ Yes - to a minor degree
- ◯ Unsure
- ◯ No
- ◯ Prefer not to say

only

19. Please expand on noteworthy negative situations you have encountered while engaging in health outreach. This is entirely optional, but helps us identify common experiences and pitfalls.

* 20. Finally, which of the following changes (if any) do you feel would benefit those communicating health the most? (check all that apply)

- [ ] Increased support from professional bodies
- [ ] Increased support from academic institutions
- [ ] Increased support from advocacy / healthcare bodies
- [ ] Clearer guidelines and better training on public engagement
- [ ] Improved emotional support services
- [ ] Other (Please specify) _______________
- [ ] Practical legal advice
- [ ] More proactive stances from institutions and bodies
- [ ] Dedicated funding for outreach
- [ ] Better protective mechanisms from internet / social media companies
- [ ] None of the above

For any queries, please contact Dr. David Robert Grimes at d.r.grimes@qub.ac.uk or davidrobert.grimes@oncology.ox.ac.uk

For peer review only - http://bmjopen.bmj.com/site/about/guidelines.xhtml

Experiences in public communication of medical science & health

Thank you for taking part in this survey. Results will be treated anonymously, but if you are happy for us to contact you and perhaps quote from your experiences if appropriate, please indicate this below.

21. If you would like to be kept up to date on results and follow-up, please enter your e-mail address below.

22. We'd be grateful if you'd also supply your twitter handle. This will not be shared without your consent, and is entirely optional, but may prove useful in future work estimating network reach of health information.

For any queries, please contact Dr. David Robert Grimes at d.r.grimes@qub.ac.uk or davidrobert.grimes@oncology.ox.ac.uk

For peer review only - http://bmjopen.bmj.com/site/about/guidelines.xhtml

BMJ Open

Establishing a taxonomy of potential hazards associated with communicating medical science in the age of disinformation

Journal:	*BMJ Open*
Manuscript ID	bmjopen-2019-035626.R2
Article Type:	Original research
Date Submitted by the Author:	13-Apr-2020
Complete List of Authors:	Grimes, David; University of Oxford, & Queens University Belfast<br>Brennan, Laura ; Not applicable , Not applicable (patient advocate, deceased)<br>O'Connor, Robert; Irish Cancer Society
**Primary Subject Heading**:	Public health
Secondary Subject Heading:	Medical education and training
Keywords:	PUBLIC HEALTH, INFECTIOUS DISEASES, ONCOLOGY, MEDICAL JOURNALISM, MEDICAL EDUCATION & TRAINING

SCHOLARONE™
Manuscripts

I, the Submitting Author has the right to grant and does grant on behalf of all authors of the Work (as defined in the below author licence), an exclusive licence and/or a non-exclusive licence for contributions from authors who are: i) UK Crown employees; ii) where BMJ has agreed a CC-BY licence shall apply, and/or iii) in accordance with the terms applicable for US Federal Government officers or employees acting as part of their official duties; on a worldwide, perpetual, irrevocable, royalty-free basis to BMJ Publishing Group Ltd ("BMJ") its licensees and where the relevant Journal is co-owned by BMJ to the co-owners of the Journal, to publish the Work in this journal and any other BMJ products and to exploit all rights, as set out in our licence.

The Submitting Author accepts and understands that any supply made under these terms is made by BMJ to the Submitting Author unless you are acting as an employee on behalf of your employer or a postgraduate student of an affiliated institution which is paying any applicable article publishing charge ("APC") for Open Access articles. Where the Submitting Author wishes to make the Work available on an Open Access basis (and intends to pay the relevant APC), the terms of reuse of such Open Access shall be governed by a Creative Commons licence – details of these licences and which Creative Commons licence will apply to this Work are set out in our licence referred to above.

Other than as permitted in any relevant BMJ Author's Self Archiving Policies, I confirm this Work has not been accepted for publication elsewhere, is not being considered for publication elsewhere and does not duplicate material already published. I confirm all authors consent to publication of this Work and authorise the granting of this licence.

For peer review only

Establishing a taxonomy of potential hazards associated with communicating medical science in the age of disinformation

David Robert Grimes[1,2*] Laura J Brennan[†], and Robert O'Connor[3]

1. School of physical sciences, Dublin City University, Glasnevin, Dublin 9, Ireland
2. Department of Oncology, University of Oxford, Off Roosevelt Rd, Oxford, UK
3. Irish Cancer Society, Dublin 4, Dublin, Ireland

Correspondence: davidrobert.grimes@dcu.ie / davidrobert.grimes@oncology.ox.ac.uk

Abstract

Objectives: Disinformation on medical matters has become an increasing public health concern. Public engagement by scientists, clinicians, and patient advocates can contribute towards public understanding of medicine. However, depth of feeling on many issues (notably vaccination and cancer) can lead to adverse reactions for those communicating medical science, including vexatious interactions and targeted campaigns. Our objective in this work is to establish a taxonomy of common negative experiences encountered by those communicating medical science, and suggest guidelines so that they may be circumvented.

Design: We establish a taxonomy of the common negative experiences reported by those communicating medical science, informed by surveying medical science communicators with public platforms.

Participants: 142 prominent medical science communicators (defined as having >1000 Twitter followers and experience communicating medical science on social and traditional media platforms) were invited to take part in a survey, with 101 responses.

Results: 101 responses were analysed. Most participants experienced abusive behaviour (91.9%), including persistent harassment (69.3%) and physical violence and intimidation (5.9%). A substantial number (38.6%) received vexatious complaints to their employers, professional bodies, or legal intimidation. The majority (62.4%) reported negative mental health sequelae due to public outreach, including depression, anxiety, and stress. A significant minority (19.8%) were obligated to seek police advice or legal counsel due to actions associated with their outreach work. While the majority targeted with vexatious complaints felt supported by their employer/professional body, 32.4% reported neutral, poor or non-existent support.

Conclusions: Those engaging in public outreach of medical science are vulnerable to negative repercussions, and we suggest guidelines for professional bodies and organisations to remedy some of these impacts on front-line members.

For peer review only

Strengths and limitations

- Individuals prominently involved in the communication of medical science across different media were surveyed to ascertain their experiences in public outreach.
- Participants were from around the world, but predominantly communicated in the English language.
- Self-selection bias in this survey is unavoidable, and findings cannot be taken as generalisable.
- Accordingly, survey results should only be taken as indicative of the scope of the issue at this juncture.
- Much further research is needed to ascertain how the medical community can best act to counter the rise of medical disinformation whilst protecting practitioners.

[†] Deceased 20th March 2019

For peer review only - http://bmjopen.bmj.com/site/about/guidelines.xhtml

Introduction

Despite being fundamental to societal well-being, many aspects of medical science remain poorly understood and frequently distrusted. Disinformation undermining health science and evidence-based medicine has increased markedly in the era of social media, and dangerous misconceptions abound, from perceived cancer risks and ostensible cures [1] to dangerous falsehoods about vaccination [2]. Improving public awareness and understanding of science and medicine is imperative if we are to maintain continued progress in research endeavours, and scientists, physicians, and science communicators have a crucial role to play in shaping public perceptions. Medical science is largely publicly funded, and direct communication of research with the wider public can be extraordinarily beneficial on a societal level. Accordingly, public engagement has become a prerequisite for many funding bodies. Informed engagement by patient advocates and media figures too can have marked impact on public understanding of medicine, empowering the public with facts with which to make important health decisions.

Improving public understanding of medical science is vital, as there many scenarios where public perception (or a vocal subset of that) is starkly at odds with scientific consensus. Frequently, medical science contradicts a narrative strongly held by particular groups within the wider public. For our purposes, we define a 'narrative' as a world-view or mindset shared by a given subgroup, which unifies that grouping. Narratives are often articles of faith, empowerment or comfort, frequently unsupported by available evidence or at odds with scientific consensus. For clarity, we concentrate herein on situations where there is no reputable evidence for a narrative, or where overwhelming scientific consensus is firmly against that viewpoint.

Misguided narratives can be supremely damaging, and the anti-vaccine movement is perhaps the most obvious example of this. Despite the life-saving efficacy of vaccination, opposition has existed since the time of Jenner [3]. The rise of social media has seen significant propagation of anti-vaccine narratives [4, 5, 6], driving uptake rates down and causing serious harm worldwide [7, 8]. In 2018, Europe saw the highest number of cases of measles cases in 20 years, numbering over 82,525 cases with at least 72 deaths - over fifteen-fold the figures from 2016 [9]. Such is the extent of the problem that in 2019 the World Health Organisation described vaccine hesitancy as a "Top ten threat to global health" [10]. Exposure to anti-vaccine conspiracy theory is a leading factor in parental intention to vaccinate [8], and evidence to date suggests that the deluge of vaccine disinformation across social media is extremely damaging to public understanding and health.

Other strongly-held narratives which clash with the weight of available scientific evidence include the claims propounded by the anti-fluoride movement [11, 12], the beliefs of the electromagnetic hypersensitivity movement [13], and the narratives of alternative medicine [14, 15]. Cancer patients are especially vulnerable to misinformation, and frequently targeted by charlatans and the misguided [16]. Consequences of this can be severe, with patients sometimes delaying or refusing conventional treatment. The net result of this is diminished survival statistics for those who engage with cancer pseudoscience, due to delayed treatment and sometimes rejection of conventional medicine. In some instances, subscribing to un-proven or disproven modalities could approximately half survival time [17].

While health falsehoods have always existed, the social media age has created new avenues for misinformation (misinformed advice) and disinformation (deliberate falsehoods) to propagate [16], rapidly bringing discredited ideas and dangerous pseudoscience to vast new audiences. Scaremongering stories from dubious outlets propagate more readily than reliable fact-based information from reputable sources [18, 19]. In 2016, over half of all cancer stories shared on

For peer review only

Facebook were medically unsound. Some have harnessed pseudoscience to sell questionable diets, supplements, and books, to the detriment of public understanding. Internet health guru Joseph Mercola, for example, made over $7 million in 2010 alone, proffering dubious treatments and advice [20], including denigration of conventional therapies for cancer. Mercola is far from unique, and such proclamations have huge potential for patient harm.

To overcome this challenge, public outreach by scientists, physicians, and evidence-based health advocates must be a crucial element to counter damaging fictions and empower our community with evidence-based information. A physician's recommendation, for example, is central to parental decisions to vaccinate [21]. Addressing patient concerns improves public health, and personal engagement by researchers and physicians can have a positive impact on public perception. Patient advocates and media figures have substantial ability to shift public perception; after Ireland saw HPV vaccine uptake drop from 87% to 51%, an alliance of healthcare professionals, researchers, and patient advocates were instrumental in countering the dominant falsehoods, and Ireland has seen a dramatic recovery in vaccine uptake rates [22]. To make inroads against the deluge of dubious health claims to which we're subjected, it's vital that scientists, clinicians, and patient groups must be on the vanguard of efforts to counter-act misinformation.

Those engaging in public outreach, however, often encounter enmity for publicly advocating scientific evidence. Scientific consensus often runs contrary to deeply-held beliefs, leading to certain groups attempting to undermine legitimate public scientific discourse. Motivations for this are multi-faceted, often depending on very specific circumstances. Conspiratorial thinking underpins many narratives, and those attempting to communication science are often vilified as "shills", or agents of a nebulous "Big Pharma". The phenomenon of identity protective cognition is also commonly encountered [23] and narrative believers frequently attacking those who cast doubt on their beliefs. Even when handled with sympathy and compassion, professional and patient advocates who challenge misconceptions can become targets for certain individuals and groups. These negative responses can range from verbal abuse to coordinated harassment campaigns, and even violence.

Aside from being deeply unsettling, such responses can cause professional and personal problems for those targeted. With the increasing emphasis on public engagement and ubiquity of the internet, this subject warrants urgent consideration, as there are currently few clear guidelines for researchers, clinicians, or patient advocates engaging in outreach work. Nor indeed is there a unified understanding of how adverse effects can manifest, and institutions and professional bodies are typically ill-equipped or muted in their support. This leaves those in the public eye or studying contentious topics vulnerable to vexatious complaints and even physical harm. Without awareness of this reality, a less than ideal situation where professional bodies can potentially be weaponised against researchers can too easily emerge. Accordingly, the aim of this work is to ascertain the typical experiences of those communicating medical science and identify how negative impacts might be counteracted.

Figure 1 A non-exhaustive taxonomy of negative experiences encountered by individuals engaging in public communication of health science. Subsections discussed in text.

For peer review only

Methods

Sample recruitment and selection criteria

The main recruitment fora for this study were online discussion groups for physicians, scientists, and patient advocates communicating aspects of medical science to the general public across social and traditional media. At the height of the Irish HPV vaccine confidence crisis [22], several physicians and scientists (based in Ireland and the UK) in these groups sought advice for negative experiences, including threats and malicious complaints to their employers and professional bodies, all of which were eventually dismissed. Group members across Europe echoed similar experiences in communicating vaccination science and in other health issues, and almost identical adverse reports came from colleagues across the Americas and Africa. Informal interviews were conducted on foot of this in these fora to identify common issues, as to the authors' knowledge there is no existent literature on the topic.

Based on these interviews and related fora discussions, a survey was created, including free-form sections where subjects were free to expand on their own experiences. The wording of this survey is included in the supplementary material. The participant selection criteria were specifically for those communicating medical science both on social media (defined as having over 1000 followers on twitter) and in mainstream channels (defined as invited appearances on public television, radio, and / or in the form of newspaper articles & invited comment). With this participant selection criteria, 142 individuals world-wide (from across Europe, America, Africa, and Asia) working predominantly in the English language were identified and invited to partake, of whom 101 (71.1%) responded. This survey was undertaken for indicative purposes and was collected from a non-randomised group with no expectation of transferable findings. Accordingly, the Health Research Authority (HRA) decision tool (online at http://www.hra-decisiontools.org.uk/research/) indicated specific ethical approval was not required, with the research governance body of Queen's University Belfast (the lead author's primary affiliation at the time) confirming ethical approval for the survey was not required. In all cases, informed consent was sought and obtained prior to subjects partaking, with all data appropriately anonymised. Subject details are given in table 1.

Patient and Public Involvement

As patient advocates play a substantial role in combatting misinformation on medical issues, several who met the inclusion criteria were invited to take part, with 15.8% of respondents being patient advocates. One co-author of this work (LBJ) was a prominent patient advocate.

Table 1: Participant details

Inclusion and completion	Invited to take part (N = 142)<br>Total Completed (N = 101)
Gender	Female (N=55)<br>Male (N=44)<br>Non-Binary/Undisclosed (N=2)
Affiliation	University/Medical Centre (N=52)<br>Unaffiliated (N=26)<br>Media organisation (N=20)<br>Charity (N=11)<br>Political organisation (N=4)
Profession	Medical Professional (N=23)<br>Scientist/Researcher (N=20)<br>Science communications (N=16)<br>Patient Advocate (N=16)<br>Health Policy (N=5)

For peer review only

Years active	Average: 10.7 years (Range 2-30 years)

Table 2 A non-exhaustive taxonomy of common adversarial tactics

**Misrepresentation of scientific evidence / expertise**		
Straw-manning	Misrepresenting scientific arguments to make them amenable to ridicule or attack, and to deflect or obscure evidence that undermines a particular narrative.	*"Mercury is toxic, yet scientists put it in vaccines!"* - This statement belies importance of dose and ignores the fact there is no evidence for harm from thimerosal in vaccines.
Cherry-picking / Quote-mining	Selective, manipulative filtering of scientific evidence or expert statements, taken out of context to undermine evidence-base or promote a narrative.	*"THC kills cancer, but doctors don't want you to know about cannabis cures."* - THC can kill cells in a petri dish, but killing plated cells is entirely different from treating human cancer.
Shifting the burden of proof	Insisting the onus is on scientists to 'disprove' claims rather than offering any evidence or rationale for assertions made.	*"GMOs are toxic, and scientists should prove us wrong"* - This assertion is untrue, and onus lies on those making the claim to proffer evidence for it.
**Discreditation attempts**		
Questioning qualification	Casting doubt on one's ability to question claims at hand. Typically, ostensible scepticism is not extended to claims supportive of the narrative.	*"This patient advocate isn't an expert, so they can't say this vaccine is safe!"* - One does not need to be an expert immunologist in this case to accurately reflect medical consensus.
Alleging vested interests	Claims that the speaker is compromised due to some apparent conflict of interest or that experts are otherwise lacking impartiality.	*"Who's paying you to say this?"* - Unsubstantiated allegation to deflect from absence of evidence for a narrative or claim.
Asserting Conspiracy theory	Allegations that the scientist, physician, or patient advocate is part of some conspiracy to suppress the truth or spread false information, either as a pawn or an active player.	*"She's part of a pharma cover-up to suppress natural cancer cures!"* - Appeals to conspiracy theory function to distract from lack of evidence.
**Dubious amplification of pseudoscientific narratives**		
Media Targeting	Targeting traditional or online media outlets to amplify dubious narratives, typically by-passing gatekeepers (science / health journalists etc) who would otherwise be more likely to spot pseudoscience.	Pitching dubious health claims to journalists as human interest stories - This approach was successfully used by anti-vaccine activists to push the dis credited link between autism and the MMR vaccine between 1998-2000.
Astroturfing / sock-puppeting	Use of fake social media accounts / fictitious pressure groups to provide an illusion of a wider grass-roots support for a particular narrative	Example: Accounts which spring up once an initial anti-fact site is blocked but which include misinformation consistent with the originator's initial social media accounts.
Celebrity endorsement	Celebrities and influencers can have disproportionately large impact on the perception of public even in areas where they have no relevant expertise or knowledge.	There are numerous examples of this, especially in relation to anti-vaccine activism, including actors and models being cited for their purported knowledge of complex health issues.
**Malicious complaints / abuse of regulatory frameworks**		
Poisoning the well / Smear campaigns	The spreading of malicious claims regarding an individual's professional or personal conduct to undermine them or discourage others from engaging with them.	*"I've heard that doctor abuses patients"* - Inflammatory slurs such as these are designed to discredit, and are not in any way substantiated, but calculated to invoke disgust or contempt.
Vexatious complaints to employers	Making calculated complaints to one's employer or threatening to do so in order to intimidate them into silence.	Exaggerated/misleading accounts of interactions with public advocates and demands to censure them, typically aimed at an individual's university or employer.
Vexatious complaints to regulatory bodies	Abusing procedures of professional bodies to target a researcher / medic who presents a challenge to a narrative. These may also include unwarranted freedom of information requests or vexatious parliamentary questions.	Registering complaints with a medical regulatory body against a doctor for their advocacy of evidence-based positions. Physicians especially vulnerable, as typically all complaints must be investigated, regardless of merit.
**Intimidation**		
Harassment / abuse	Harassment can take many forms, and personal abuse is perhaps most common. Threats (implied and direct) are often employed.	Abusive language made publicly or in direct messages, and posting of private contact details, phone numbers, addresses etc.
Legal threats	Legal notices and mechanisms can also be utilised to silence researchers questioning a narrative, from cease and desist notices to defamation claims.	Threatening to bring an advocate to court for alleged defamation, also used judiciously to limit independent investigation on pseudoscientific narratives
Physical intimidation	Implicit or explicit threats of physical violence is an u~~U~~nfortunately, potent method of intimidating scientists into silence. This includes threats of physical violence or rape (the latter usually directed at female discussants)	Stalking of private abodes, explicit threats, or actions like spitting etc. There are instances of security being required for scientific meetings on publicly contentious subjects, due to implications of or threats of violence.

For peer review only

Results

142 individuals were approached to undertake the survey, with 101 responding (response rate: 71.1%). In addition to survey questions on known problems, participants were also invited to expand on noteworthy negative situations they may have encountered while engaging in health outreach. This section was entirely optional, and 53 participants (52.5% of subjects) opted to share their experiences. Example responses are shown below; please note that some responses have been edited or partially redacted to exclude potentially identifying information and preserve anonymity.

"Accusations - including by one Senator - that [we are] uncaring, dismissive, neglectful, arrogant, or paid by pharma companies when advocating for vaccines." (Misrepresentation)

"I find my expertise is questioned - this often seems to be when men find it difficult to accept women with intelligence and qualifications. Sexist insults are a typical go-to response." (Discreditation)

"The worst one that hurt me professionally and personally was that activists gathered my emails using [Freedom of Information Requests] and handed chosen packets of them with a story to different reporters." (Misrepresentation/Discreditation/Dubious Amplification)

"Persistent negative comments on twitter; usually it doesn't last long but it can feel very intense while it's happening!" (Intimidation)

"I have been served with a SLAPP lawsuit in order to silence my outreach work. Frequently receive harassing emails, malicious comments made on blog." (Malicious Complaints)

"Social media co-ordinated intimidation, implied threats of legal action (for defamation). Mocking, undermining, condescension and attacks for being an industry shill, although.. I am just a patient advocate. Being called a liar, that I never had cancer, that I deserved cancer due to my attitude, that I have been mutilated by conventional medical treatment, and that I am no longer a woman (having had mastectomy for cancer). That my cancer will return and I deserve that." (Dubious Amplification / Misrepresentation/Discreditation)

"I have had anti-vaccine organizations and individuals attempt to prevent my public appearances and have been the subject of numerous online smear campaigns accusing me of being 'a shill for Big Pharma' etc." (Discreditation/Dubious Amplification)

"Those who attack me very frequently try to do it by targeting me at my job, sending bogus complaints to my bosses and the university. From my observation, that is the go-to attack, the first thing these groups do." (Malicious Complaints)

"I had to contact the police, who visited the person who was harassing me. I also involved social services. We bought a CCTV to monitor our front door after a strange envelope was hand delivered. The person involved has targeted several people before and continues to target individuals who advocate vaccination." (Intimidation)

"Abuse and accusations of corruption are the most common adverse reaction I get. Sometimes a particular group petition one's employer and try to create trouble for them. I have been lucky in the past when this happened to have had supportive universities who appreciate my outreach work. I have in the past had slightly unhinged individuals writing rambling, implicitly threatening letters to my office which ultimately required police intervention." (Discreditation/ Malicious Complaints/Intimidation)

For peer review only

"The worst are gendered insults (being called a cunt, etc.) and rape/death threats. I have had one empty legal threat that was widely publicized." (Intimidation/Malicious Complaints)

"Regular threats to sue for defamation." (Malicious Complaints)

"Attempts to get me fired, public records act requests for emails, verbal attacks on my children." (Malicious Complaints, Intimidation)

"One of the most unpleasant things is that certain people or groupings will use very underhanded tactics to respond to perceived criticism. If they can't refute the science, it isn't uncommon for them to go after you personally, alleging all manner of things to anyone who'll listen; that you're incompetent, or unethical, or perverted. It seems they throw things wildly to see what sticks, but it can be extraordinarily unpleasant to endure." (Dubious Amplification/Discreditation)

"My main concern has been obsessed individuals who declare their enmity and seem to be unconstrained by civil norms." (Intimidation)

"Homeopathy advocates looked up my Linkedin profile and called my employer to complain about my comments on the radio. My employer did not support me and I ended up having to stop the activity I had been planning." (Misrepresentation/Malicious Complaints)

"Being threatened with physical violence" (Intimidation)

"A delusional supporter of [an individual] I wrote about accused me and my lawyer of stalking him and killing his in-laws. He sent accusing emails to the faculty of my school and all the police departments in my state. [They] also accused me of being a terrorist and complained about me to the FBIs Terrorism Joint Task Force. That gave me many nervous, sleepless nights." (Discreditation/Malicious Complaints)

"Death threats received, employer unhelpful, sorted myself" (Intimidation)

"I haven't experienced many negative encounters because I would say I am only lightly involved in public engagement. However the reason I don't become more heavily involved in this area is fear of this kind of abuse and vexatious complaints to my employer or regulatory body." (Malicious Complaints)

Based upon this and survey responses, a non-exhaustive taxonomy was constructed detailing common experiences of those communicating medical science to the public. While non-exhaustive, it forms a useful basis for more systematic investigation. Adversarial experiences in communicating medicine were broadly stratified into five distinct classes, illustrated graphically in figure 1, with these sub-types detailed in table 2.

Participant details are given in table 1. Topics covered, channels of engagement, and fora for abusive interactions are depicted in figure 2, informed by survey questions 4, 5, and 16. Twitter is disproportionately represented, as prominence on that platform was part of the selection criteria. Other fora cited included books, documentaries, newspapers, podcasts - and in one instance criticism under parliamentary privilege. The vast majority of those surveyed (N = 94, 93.1%) reported being the recipient of personal abuse of professional smears in the course of their outreach efforts. A majority (N = 70, 69.3%, survey question 13) had experienced targeted abuse from at least one particular grouping. The most common groupings for negative reactions were anti-vaccine and alternative medicine groups respectively (N = 43 each, 42.6%) followed by dietary advocates (N = 26, 25.7%),

For peer review only

’wellness’ groups (N = 17, 16.8%), religious and chronic illness groups (both N = 15, 14.9%), anti-fluoride and autism focused groups (both N =12, 11.9%). Others cited by 3 or less respondents included electromagnetic hypersensitivity factions, conspiracy theorists and anti-GMO organisations.

Figure 3 depicts types of experiences reported by participants, ranging from the relatively minor to the severe, informed by survey responses to questions 11, 12, and 14. Of participants surveyed, a majority (N = 63, 62.4%) reported fallout from public engagement had caused them at least some negative mental health sequelae, including depression, anxiety, and stress. Most of this was reported as minor, but considerable or significant mental health ramifications were reported by 15 respondents (14.9%). Of the participants, 20 (19.8%) were obligated to seek police advice/legal counsel as a direct result of targeted actions associated with their outreach work. Of those receiving vexatious complaints (N = 39, 38.6% of all respondents), most (67.6%) felt supported or well supported by their institution, employer, or professional body, with whilst 16.2% deemed support to be neutral, with an equal number (16.2%) feeling poorly or entirely unsupported. Predictably perhaps, gender specific abuse was far more likely to be directed at women (40% of female respondents) than men (6.8% of male respondents), with this difference being highly significant ($p << 0.001$, calculated by Welch’s t-test)

Table 3 depicts frequency of different experiences (positive and negative) reported by respondents, taken from data in survey question 8. In response to survey question 9, 29.7% (N=30) responded that they found outreach largely rewarding, 38.6% rewarding (N=39), 29.7% mixed (N=30), and ~2% not very rewarding (N=2). Changes respondents felt would most improve outreach work are depicted in figure 4 (from survey question 20). Free-form responses to this question included; improving the media’s scientific literacy (false balance and the platforming of anti-science views were repeatedly mentioned); the establishment of legal defence funds; better coordination of professional bodies; robust infrastructure on social media to report disinformation, and better support from police organisations.

Table 3: Frequency of experiences with outreach

Statement	**Always**	**Frequently**	**Sometimes**	**Infrequently**	**Never**	**Unsure**
Engagement is mutually informative	6	31	52	10	2	0
Engagement changes minds & informs	1	22	64	7	1	6
My contributions are welcome & appreciated	1	56	39	3	0	0
My efforts contribute to public understanding	4	47	49	1	0	0
My efforts are taken in good faith	3	51	38	6	0	3
My efforts feel futile	1	9	50	34	7	0
Engagement takes a toll on mental health	2	12	44	26	16	0

Figure 2 (a) Topics covered by participants (b) channels of engagement for subjects surveyed (c) Fora for negative interactions

Figure 3 Proportion of negative experiences recorded including (a) abuse experienced (b) violence and intimidation (c) vexatious complaints.

Figure 4 Changes respondents deemed most likely to benefit medical science communication the most.

For peer review only

Discussion

In a globalised information age, medical science can appear disconnected and aloof from those it serves to help. Educational and professional bodies (including universities and medical centres) have a unique societal role to inform their peers and public on evidence-based medicine, and a responsibility to adjust to modern communications realities. We can collectively no longer remain on the fence in supporting health information advocacy. While being mindful not to over-infer from our survey, we can use it as a basis to make some suggestions. It is vital to have proactive strategies in place to support those engaging in medical outreach, and to maintain a high calibre for public discussion. It is also crucial that those engaging in outreach are cognizant of the potential pitfalls, and afforded ample support. Given the gendered nature of much of the abuse reported, it seems likely that the hostile environment encountered online could dissuade many talented female communicators from engaging fully, to focus on but one example. It is also important to note that despite the sometimes fraught nature of medical science outreach, a majority of respondents (68.2%, N = 69) found the undertakings rewarding or very rewarding. This is encouraging, but it is crucial we are aware too of the adverse effects that can arise from communicating medical science, many of which are outlined in this manuscript.

One potential weakness of the survey is the potential for ambiguous definition. Alternative medicine, for example, is typically defined as ostensible medical interventions for which there is insufficient or disconfirmatory evidence; for example, the National Science Board define it as referring to "all treatments that have not been proven effective using scientific methods". As no specific definition was given in the survey text, it is possible respondents substituted their own meaning to some extent. As those surveyed were drawn from science communicators with significant media profiles however, it might be expected that their definitions were more unified than a typical respondent might be. There is also some unavoidable ambiguity with terms such as "abuse" and "smears". There is a level of subjectivity to these terms, which respondents were left to define themselves. This renders the responses potentially subjective, although the free-form responses do indicate behaviours that could be seen as objectively abusive.

There is also a serious point that must be at least considered – that advocates for medical science may on occasion engage in ill-advised tactics or unhelpful rhetoric. Nor does one's expertise render them infallible, and it is certainly possible that advocates for science might sometimes engage in a counterproductive fashion. To ascertain this requires some context and nuance, especially for academic and medical institutions whose staff might be the subject of complaints. But rather than be reactionary, it is imperative that bodies and institutions have robust and considered policies for dealing with issues that might arise. The benefits of this are two-fold; firstly, so that errant behaviour by members can be corrected. But equally importantly, cognisance of the reality of vexatious complaints also means that bodies and institutions can implement measures to ensure that their procedures cannot be weaponised by malicious actors. Based on the feedback to this survey and wider discussion on the topic, we offer the following suggestions to employers and professional bodies whose members might engage in the communication of medical science. While by no means comprehensive, the following guidelines might be beneficial towards establishing policy for dealing with issues that can arise.

Suggested guidelines for professional bodies and employers

1. Educational/professional organisations must recognise a commitment to support evidence-based actions by their members. This may require oversight of such activities and investment in the governance/training resources to protect members willing to act as advocates.

2. Institutions and professional bodies should have robust measures in place to oversee communication activities associated with their members, and to make assessments as to whether individuals are communicating established facts in good faith or are contributing to undermining of facts with potential legal/reputational damage.

3. When institutions receive complaints regarding members, the subject must be afforded presumption of innocence rather than being served with reactionary and inflexible procedures, lest the institution might become an unwilling tool against science.

4. In case of disputes and complaints, competent and impartial individuals should be engaged to independently assess complaints, cognisant of vital background and context.

5. Coordination between press offices and those engaged in outreach would improve communication, pre-emptively identifying those likely to be targets for malicious tactics.

6. Support for those identified as being on engagement frontlines should be maintained, with clear legal advice/institutional support for targeted members.

7. Organisations must be vocal in supporting public-facing members, willing to issue strong rebuttals of vexatious complaints against individuals.

8. Professional bodies and employers should strive to promote both scientific freedom of speech and to champion evidence-based advocacy.

9. When possible, those expected to engage in outreach should be trained in methods that reduce opportunities for personal and professional attacks.

Conclusions

The question of how we best communicate health science in the modern era is an area where more research is urgently required, especially on the role of social media, and optimum ways physicians, researchers, and other public-facing figures can promote good medical science and mitigate falsehoods. The suggestions herein ought to be taken as a starting point, with discussion evolving as improved evidence materialises. There are wider problems implicit in all this that those communicating science cannot tackle in isolation; social media regulation particularly is a serious issue, both in regards to the spreading of misinformation/disinformation, and with respect to procedures preventing the potential weaponisation of social media platforms. Social media platforms must ultimately be made answerable to regulatory over-sight, just as every other important aspect of life is; claims of innocence are unconvincing when their business model is so clearly dependent on advertising engagement at the cost of lives. The problem of poor reporting and false balance [24] in conventional media outlets also must be considered, and there is significant scope for scientists and doctors to contribute to policy in these areas. There is ample evidence that physicians and scientists have an important role to play in combatting health disinformation, as has recently been argued by one of the authors in relation to vaccination for British Medical Journal opinion [25]. But equally, it is crucial that those engaging in this vital work have the requisite support from their institutions, so that deleterious consequences of laudable outreach work might be circumvented. It is increasingly clear that disinformation about medicine and illness has become ubiquitous, with severe consequences for both our collective health and public understanding of medical science. Scientists and physicians must be at the vanguard of the pushback against these dangerous falsehoods – our societal well-being depends upon it.

For peer review only

Electronic Supplementary Material

A full version of the questionnaire and further analysis can be found in the electronic supplementary material.

Acknowledgements

The authors would like to thank the scientists, physicians, and patient advocates who gave their time to discuss the issues they face in communicating science, and for sharing their insights, and the reviewers for their useful recommendations. In particular, the authors would like to profoundly thank Dr Laura J Brennan for her invaluable input; Laura was present and helped conceive the concept from which this paper sprung. She was a passionate advocate of evidence-based science communication and especially for the HPV vaccine, championing it despite enduring a life-limiting and difficult cervical cancer diagnosis. Following a confidence crisis in the vaccine in Ireland driven by anti-vaccine activism, her tireless work was a substantial factor in reversing the damage wrought by disinformation. Laura passed away 20th March 2019, but while we have lost a friend and colleague, hers is a legacy that will resonate for generations. Laura embodied precisely how vital health communication is to promoting public health; Ireland, and the world, owe her a huge debt, and we dedicate this work to her memory.

Contributor statement

DRG, LJB & ROC conceived the concept for this paper, and worked on survey design. DRG & ROC performed the analysis and wrote the manuscript.

Competing interests

The authors declare that they have no competing interests.

Funding statement

The authors declare that they have no specific funding for this work.

Data Sharing

Full survey forms available in electronic supplementary material.

References

1. Shahab, L., McGowan, J.A., Waller, J., Smith, S.G.: Prevalence of beliefs about actual and mythical causes of cancer and their association with socio-demographic and health-related characteristics: Findings from a cross-sectional survey in england. European Journal of Cancer 103, 308{316 (2018)

2. Kata, A.: A postmodern pandora's box: anti-vaccination misinformation on the internet. Vaccine 28(7), 1709-1716 (2010)

For peer review only

3. Poland, G.A., Jacobson, R.M.: The age-old struggle against the antivaccinationists. New England Journal of Medicine 364(2), 97{99 (2011)

4. Zimmerman, R.K., Wolfe, R.M., Fox, D.E., Fox, J.R., Nowalk, M.P., Troy, J.A., Sharp, L.K.: Vaccine criticism on the world wide web. Journal of medical internet research 7(2), 17 (2005)

5. Kata, A.: Anti-vaccine activists, web 2.0, and the postmodern paradigm{an overview of tactics and tropes used online by the anti-vaccination movement. Vaccine 30(25), 3778{3789 (2012)

6. Grimes, D.R.: On the viability of conspiratorial beliefs. PloS one 11(1), 0147905 (2016)

7. Goertzel, T.: Conspiracy theories in science. EMBO reports 11(7), 493{499 (2010)

8. Jolley, D., Douglas, K.M.: The e ects of anti-vaccine conspiracy theories on vaccination intentions. PloS one 9(2), 89177 (2014)

9. Thornton, J.: Measles cases in Europe tripled from 2017 to 2018. British Medical Journal Publishing Group (2019)

10. Organization, W.H., et al.: Ten threats to global health in 2019. 2019. Retrieved January (2019)

11. Freeze, R.A., Lehr, J.H.: The Fluoride Wars: How a Modest Public Health Measure Became America's Longest-Running Political Melodrama. John Wiley & Sons, ??? (2009)

12. Grimes, D.R.: Commentary on "are fluoride levels in drinking water associated with hypothyroidism prevalence in england? a large observational study of gp practice data and fluoride levels in drinking water". J Epidemiol Community Health 69(7), 616{616 (2015)

13. Grimes, D.R., Bishop, D.V.: Distinguishing polemic from commentary in science: Some guidelines illustrated with the case of sage and burgio (2017). Child development 89(1), 141{147 (2018)

14. Singh, S., Ernst, E.: Trick or Treatment: The Undeniable Facts About Alternative Medicine. WW Norton & Company, (2008)

15. Grimes, D.R.: Proposed mechanisms for homeopathy are physically impossible. Focus on Alternative and Complementary Therapies 17(3), 149{155 (2012)

16. The, L.O.: Oncology," fake" news, and legal liability. The Lancet. Oncology 19(9), 1135 (2018)

17. Johnson, S.B., Park, H.S., Gross, C.P., James, B.Y.: Complementary medicine, refusal of conventional cancer therapy, and survival among patients with curable cancers. JAMA oncology 4(10), 1375{1381 (2018)

18. Vosoughi, S., Roy, D., Aral, S.: The spread of true and false news online. Science 359(6380), 1146{1151 (2018)

19. Brady, W.J., Wills, J.A., Jost, J.T., Tucker, J.A., Van Bavel, J.J.: Emotion shapes the diffusion of moralized content in social networks. Proceedings of the National Academy of Sciences 114(28), 7313{7318 (2017)

20. Smith, B.: Dr. mercola: Visionary or quack? Chicago (2012)

21. Rosenthal, S., Weiss, T.W., Zimet, G.D., Ma, L., Good, M., Vichnin, M.: Predictors of hpv vaccine uptake among women aged 19-26: importance of a physician's recommendation. Vaccine 29(5), 890{895 (2011)

22. Corcoran, B., Clarke, A., Barrett, T.: Rapid response to hpv vaccination crisis in ireland. The Lancet 391(10135), 2103 (2018)

23. Kahan, D.M.: Misconceptions, misinformation, and the logic of identity-protective cognition (2017)

24. Grimes, D.R.: A dangerous balancing act. EMBO reports (2019)

25. Grimes, D.R.: "David Robert Grimes: Vaccines-How Can We Counter Misinformation Online?" The BMJ, 7 Nov. 2019, blogs.bmj.com/bmj/2019/11/06/david-robert-grimes-vaccines-how-can-we-counter-misinformation-online/.

For peer review only

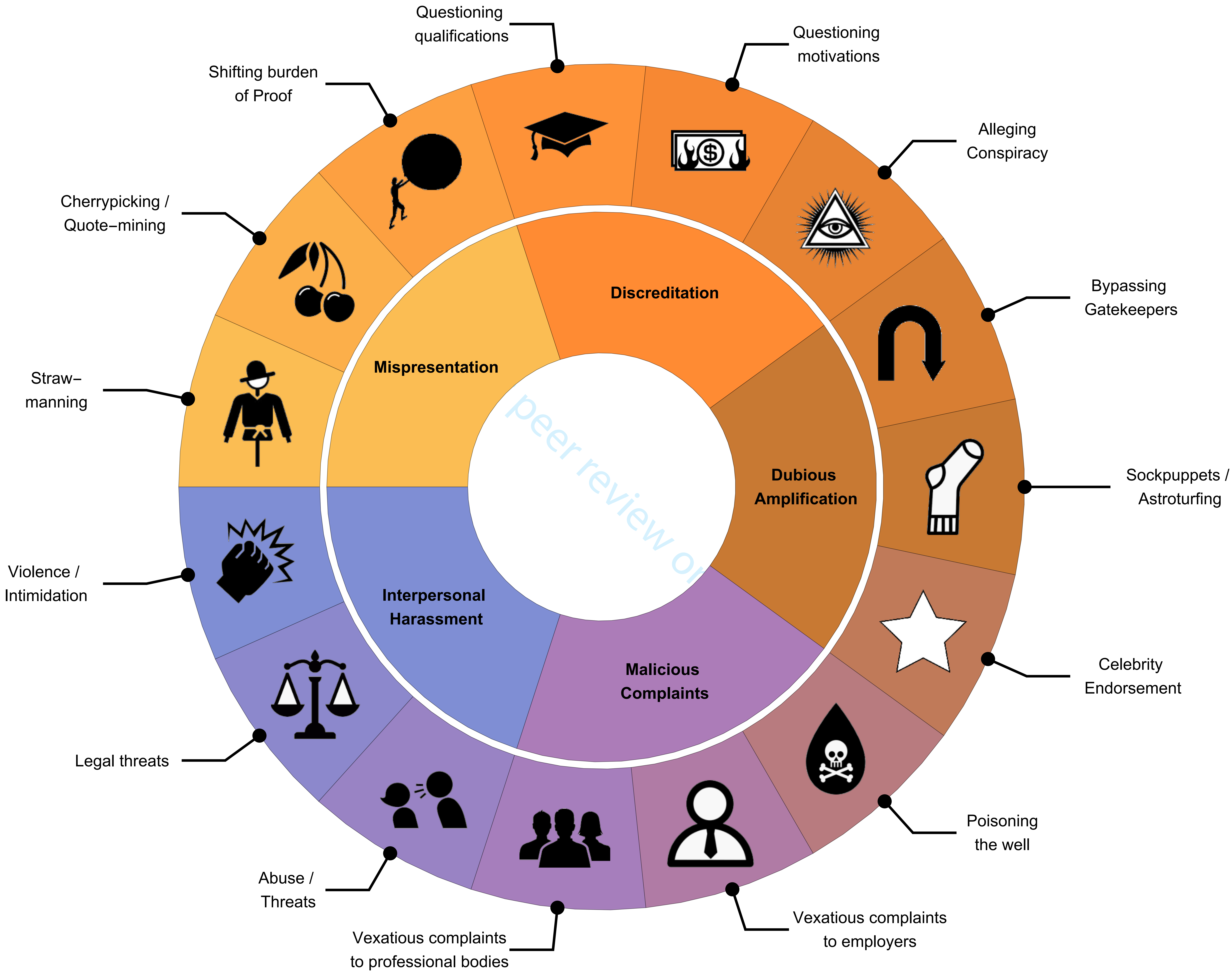

peer review only

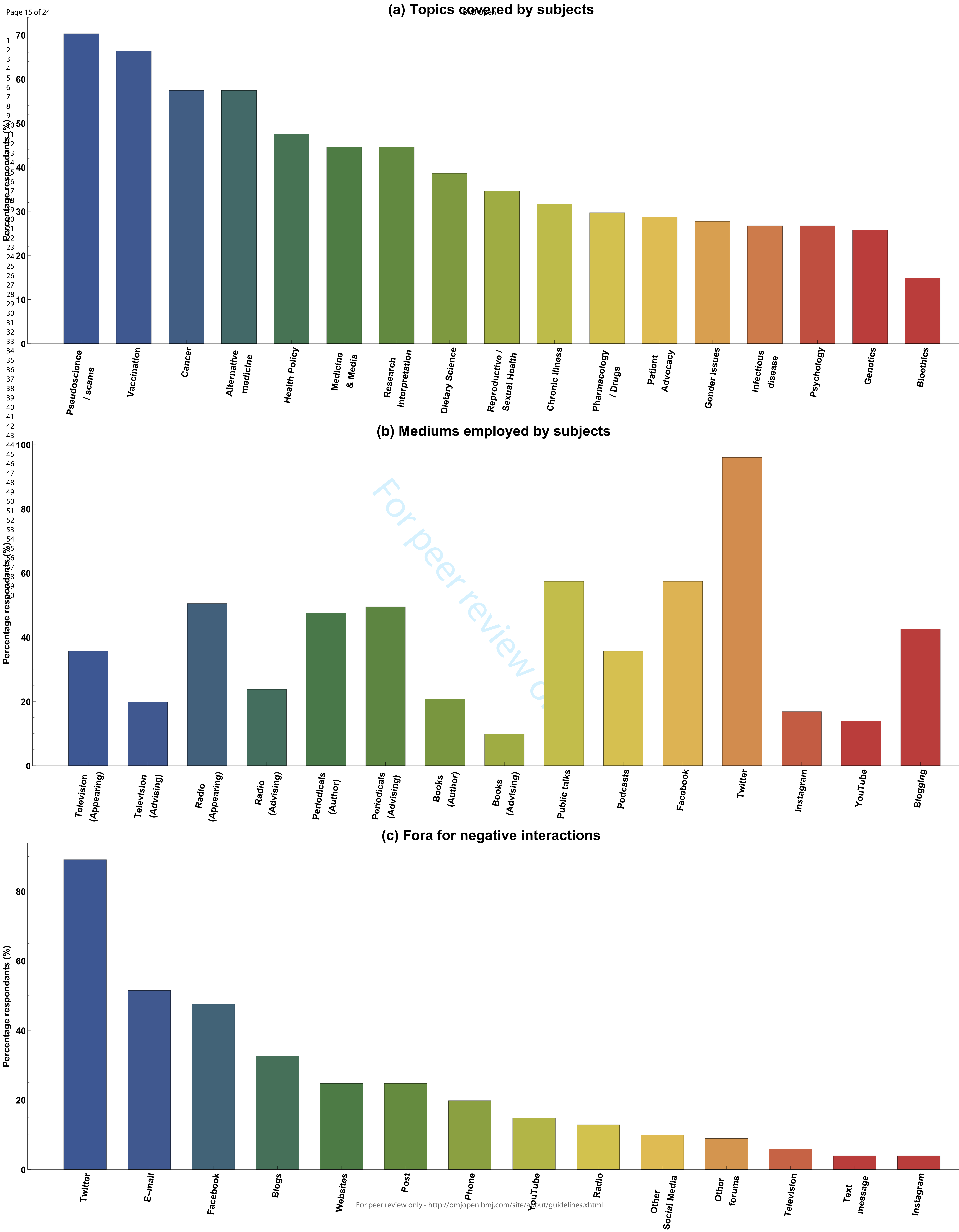

For peer review only

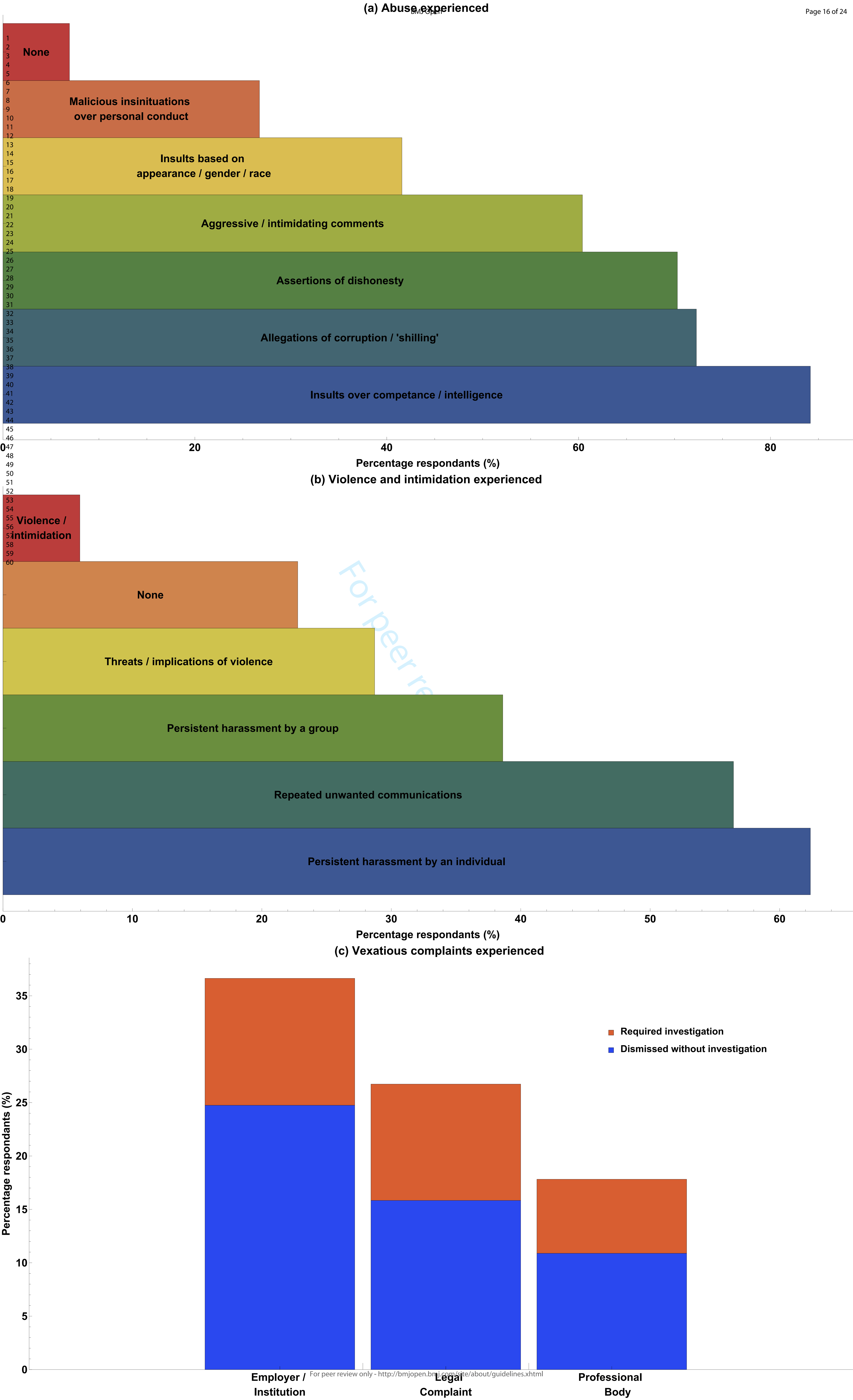
(a) Abuse experienced
None
Malicious insinituations over personal conduct
Insults based on appearance / gender / race
Aggressive / intimidating comments
Assertions of dishonesty
Allegations of corruption / 'shilling'
Insults over competance / intelligence
0
20
40
60
80
Percentage respondants (%)
(b) Violence and intimidation experienced
Violence / intimidation
None
Threats / implications of violence
Persistent harassment by a group
Repeated unwanted communications
Persistent harassment by an individual
0
10
20
30
40
50
60
Percentage respondants (%)
(c) Vexatious complaints experienced
Percentage respondants (%)
0
5
10
15
20
25
30
35
Required investigation
Dismissed without investigation
Employer / Institution
Legal Complaint
Professional Body

For peer re

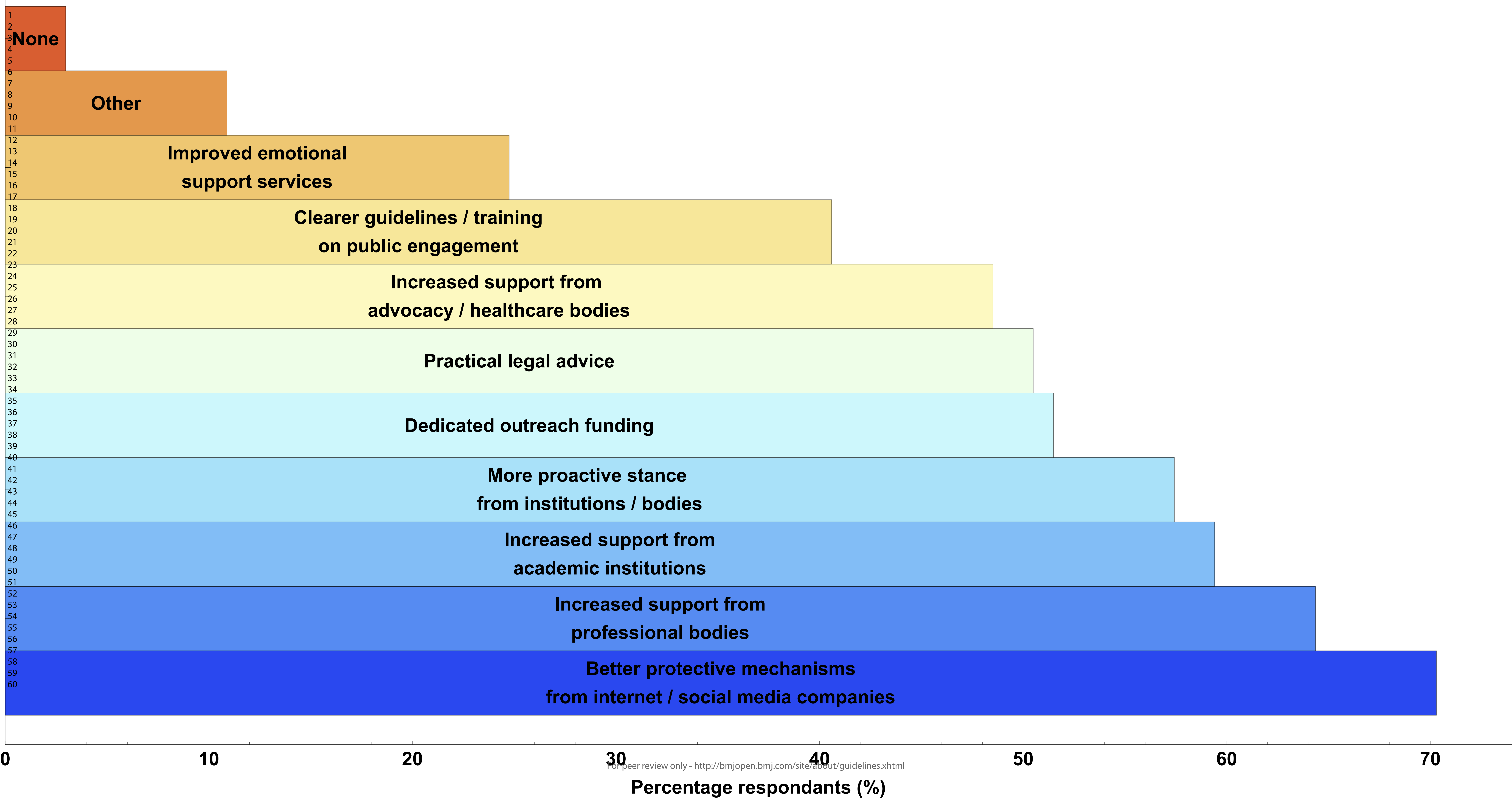

For peer review only - http://bmjopen.bmj.com/site/about/guidelines.xhtml

* Required Information

Experiences in public communication of medical science & health

This survey is part of a research project that aims to better understand the experiences of medical professionals, scientific researchers, patients advocates, health journalists, & policy makers in communicating health issues to the public. It consists of 20 short questions, & should take 5-10 minutes to complete. All responses are anonymous, and their content will be used for research purposes only. If you might be willing to contribute more about your experiences we invite you to inform us of this on the final page of the survey. For any questions, please e-mail d.r.grimes@qub.ac.uk

*** 1. Please select that which best describes your primary role (Select one option)**

- ○ Medical Professional
- ○ Scientific Communications
- ○ Scientist / Researcher
- ○ Health Journalism
- ○ Patient Advocate
- ○ Health Policy
- ○ Other (Please specify) __________

*** 2. Please specify your gender (Select one option)**

- ○ Female
- ○ Non-Binary
- ○ Male
- ○ Prefer not to say

*** 3. Please indicate which of the following organsiations you are professionally affiliated with that are directly relevant to your outreach efforts.**

- ☐ University / research institute
- ☐ Media outlet
- ☐ Medical institute
- ☐ Political grouping
- ☐ NGO / Charity
- ☐ Non-affiliated (Patient Advocate / independent)
- ☐ Other (Please specify) ______________

*** 4. Please select the topic or topics you frequently communicate about with the public. Please note that this includes topics you might debunk or criticize. Select all that apply.**

- ☐ Vaccination
- ☐ Alternative Medicine
- ☐ Medicine in media
- ☐ Dietary Science
- ☐ Pseudoscience / scams
- ☐ Medical science
- ☐ Cancer
- ☐ Chronic illness
- ☐ Bioethics
- ☐ Health Policy
- ☐ Infectious diseases
- ☐ Interpretation of research
- ☐ Reproductive / Sexual Health
- ☐ Pharmacology / drugs
- ☐ Gender issues
- ☐ Patient Advocacy
- ☐ Psychological issues
- ☐ Genetics
- ☐ Other (Please specify) ______________

*** 5. What media do you use for public engagement? Select all that apply.**

- ☐ Television (appearances)
- ☐ Television (advising / quoted)
- ☐ Radio (appearances)
- ☐ Radio (advising / quoted)
- ☐ Newspapers / Magazines (Author)
- ☐ Newspapers / Magazines (advising / quoted)
- ☐ Books (Author)
- ☐ Books (Advising / quoted)
- ☐ Public talks
- ☐ Podcasts
- ☐ Facebook
- ☐ Twitter
- ☐ Instagram
- ☐ Youtube
- ☐ Blogging
- ☐ Other (Please specify) _______________

*** 6. Approximately how long have you been communicating medical issues to the public? Please round to the nearest whole year**
(Enter a value between 0 and 120)

____

*** 7. Please select what best describes the extent of your public engagement (Select one option)**

- ◯ I am heavily involved in public outreach
- ◯ I am considerably involved in public outreach
- ◯ I am involved in public outreach
- ◯ I am lightly involved in public outreach
- ◯ My vocation is media or outreach centred
- ◯ Other (Please specify) ___________

For any queries, please contact Dr. David Robert Grimes at d.r.grimes@qub.ac.uk or davidrobert.grimes@oncology.ox.ac.uk

Experiences in public communication of medical science & health

Part II: Engagement experiences

8. In your opinion, how frequently do the following sentiments apply to your experiences of public engagement?

	Always	Frequently	Sometimes	Infrequently	Never	Unsure
*(a) I feel my efforts contribute to public understanding	○	○	○	○	○	○
*(b) I find engagement prompts constructive and respectful discussion	○	○	○	○	○	○
*(c) Engagement is mutually informative	○	○	○	○	○	○
*(d) My contributions are welcomed & appreciated	○	○	○	○	○	○
*(e) Engagement increases my personal understanding	○	○	○	○	○	○
*(f) I feel I correct some misconceptions	○	○	○	○	○	○
*(g) I find my engagement changes minds & informs	○	○	○	○	○	○
*(h) I consider outreach personally rewarding	○	○	○	○	○	○
*(i) My efforts feel futile	○	○	○	○	○	○
*(j) Engagement takes a toll on my mental health & well-being	○	○	○	○	○	○
*(k) I find my efforts are taken in good faith by others	○	○	○	○	○	○

*** 9. Please indicate whether you have experienced any of the following negative experiences during your engagement efforts. Select all that apply. Note that for the purposes of this survey, vexatious complaints are defined as those raised chiefly to harass or intimidate.**

- ☐ Personal abuse
- ☐ Personal smears
- ☐ Professional smears
- ☐ Physical threats
- ☐ Threats of sexual violence
- ☐ Harassment from individuals
- ☐ Coordinated harassment from specific groups
- ☐ Questioning of one's motivations
- ☐ Other (Please specify) _______________
- ☐ Sexist or gender-specific abuse
- ☐ Racist of ethnic-specific abuse
- ☐ Intimidation attempts
- ☐ Vexatious legal threats
- ☐ Vexatious complaints to employers
- ☐ Vexatious complaints to professional bodies
- ☐ Persistent unwanted communication
- ☐ No negative experiences

*** 10. How would you best describe your feelings about your outreach experiences to date? (Select one option)**

- ○ Largely rewarding
- ○ Rewarding
- ○ Mixed
- ○ Not very rewarding
- ○ Not at all rewarding
- ○ Other (Please specify) ___________

For any queries, please contact Dr. David Robert Grimes at d.r.grimes@qub.ac.uk or davidrobert.grimes@oncology.ox.ac.uk

For peer review only - http://bmjopen.bmj.com/site/about/guidelines.xhtml

Experiences in public communication of medical science & health

*** 11. If you have been the recipient of abuse or personal / professional smears stemming from your engagement, please indicate which of the following you've experienced (check all that apply)**

- ☐ Aggressive or intimidating comments
- ☐ Insults based on appearance, race, or gender
- ☐ Malicious comments about one's motivations / allegations of 'corruption' or 'shilling'
- ☐ Malacious comments about one's personal or sexual conduct
- ☐ Other (Please specify) ______________
- ☐ Assertions that you are dishonest or deliberately misleading
- ☐ Insults about your professional competence or intelligence
- ☐ I have not experienced any of the above

*** 12. Please indicate whether you have been subjected to any of the following (check all that apply)**

- ☐ Threats or implications of physical violence
- ☐ Physical violence or intimidation
- ☐ Spreading of malicious rumours
- ☐ Repeated unwanted communications
- ☐ Other (Please specify) ______________
- ☐ Persistent trolling / harassment by an individual
- ☐ Persistent trolling / harassment by a group
- ☐ None of the above

*** 13. If you have been the victim of targeted abuse for your outreach work by a group or community, what best describes that grouping? Choose all that apply if relevant**

- ☐ Anti-vaccine groups
- ☐ Dietary advocates
- ☐ Chronic illness groups
- ☐ Anti-fluoride groups
- ☐ Electromagnetic hypersensitivity groups
- ☐ Other (Please elaborate as appropriate) ______________
- ☐ Religious groupings
- ☐ Alternative medicine advocates
- ☐ "Wellness" groups
- ☐ Autism-focused groups
- ☐ I have not encountered this

*** 14. Please indicate whether you have experienced any of the following. Note that for the purposes of this survey, vexatious complaints are defined as those raised chiefly to harass or intimidate.**

- ☐ Vexatious complaint to employer or institution - complaint dismissed without investigation
- ☐ Vexatious complaint to employer or institution - required investigation
- ☐ Vexatious complaint to a professional body - complaint dismissed without investigation
- ☐ Vexatious complaint to a professional body - required investigation
- ☐ Vexatious legal complaint - complaint dismissed without investigation
- ☐ Vexatious legal complaint - complaint required investigation
- ☐ None of the above

*** 15. If you have experienced a vexatious complaint, did you feel supported by your institution or professional body? (Select one option)**

- ○ Highly supported
- ○ Supported
- ○ Neutral
- ○ Not well supported
- ○ Entirely unsupported
- ○ Not applicable

*** 16. If you have experienced abusive or diminishing comments, in which fora are these typically delivered or shared? (check all that apply)**

- ☐ E-mail
- ☐ Post
- ☐ Phone calls
- ☐ Texts
- ☐ Twitter
- ☐ Facebook
- ☐ Instagram
- ☐ Other (Please specify) _______________
- ☐ Youtube
- ☐ Other social media
- ☐ Blogs
- ☐ Websites
- ☐ Public television
- ☐ Public radio
- ☐ Not applicable

*** 17. Have you ever had to take legal action or consult law-enforcement officials regarding malicious communications, threats, or claims? (Select one option)**

- ○ Yes
- ○ No
- ○ Not applicable
- ○ Prefer not to say

*** 18. Have negative reactions to public engagement ever caused you mental health problems (depression, anxiety, stress, etc) or otherwise impeded your functioning? (Select one option)**

- ○ Yes - severely
- ○ Yes - considerably
- ○ Yes - to a minor degree
- ○ Unsure
- ○ No
- ○ Prefer not to say

only

19. Please expand on noteworthy negative situations you have encountered while engaging in health outreach. This is entirely optional, but helps us identify common experiences and pitfalls.

*** 20. Finally, which of the following changes (if any) do you feel would benefit those communicating health the most? (check all that apply)**

- [ ] Increased support from professional bodies
- [ ] Increased support from academic institutions
- [ ] Increased support from advocacy / healthcare bodies
- [ ] Clearer guidelines and better training on public engagement
- [ ] Improved emotional support services
- [ ] Other (Please specify) _______________
- [ ] Practical legal advice
- [ ] More proactive stances from institutions and bodies
- [ ] Dedicated funding for outreach
- [ ] Better protective mechanisms from internet / social media companies
- [ ] None of the above

For any queries, please contact Dr. David Robert Grimes at d.r.grimes@qub.ac.uk or davidrobert.grimes@oncology.ox.ac.uk

For peer review only - http://bmjopen.bmj.com/site/about/guidelines.xhtml

Experiences in public communication of medical science & health

Thank you for taking part in this survey. Results will be treated anonymously, but if you are happy for us to contact you and perhaps quote from your experiences if appropriate, please indicate this below.

21. If you would like to be kept up to date on results and follow-up, please enter your e-mail address below.

22. We'd be grateful if you'd also supply your twitter handle. This will not be shared without your consent, and is entirely optional, but may prove useful in future work estimating network reach of health information.

For any queries, please contact Dr. David Robert Grimes at d.r.grimes@qub.ac.uk or davidrobert.grimes@oncology.ox.ac.uk

For peer review only - http://bmjopen.bmj.com/site/about/guidelines.xhtml
